# *Tinospora cordifolia* as a potential neuroregenerative candidate against glutamate induced excitotoxicity: an in vitro perspective

**DOI:** 10.1186/s12906-018-2330-6

**Published:** 2018-10-01

**Authors:** Anuradha Sharma, Gurcharan Kaur

**Affiliations:** Department of Biotechnology, Medical Biotechnology lab, Guru Nanak Dev University, Amritsar, Punjab 143005 India

**Keywords:** *Tinospora cordifolia*, Neuritogenesis, Neurodegeneration, Neuroprotection, Neurotoxicity, Neuronal plasticity, Cerebellar neurons

## Abstract

**Background:**

Glutamate, the major excitatory neurotransmitter of CNS acts as a neurotoxin at higher concentrations. Prolonged activation of glutamate receptors results in progressive neuronal damage by aggravating calcium influx, inducing mitochondrial damage and oxidative stress. Excitotoxic cell death is associated with the pathogenesis of various neurodegenerative disorders such as trauma, brain injury and neurodegenerative diseases. The current study was designed to investigate the neuroprotective and neuroregenerative potential of *Tinospora cordifolia* against glutamate-induced excitotoxicity using primary cerebellar neuronal cultures as a model system.

**Methods:**

Monosodium salt of glutamate was used to induce neurotoxic injury in primary cerebellar neurons. Four extracts including Hexane extract, Chloroform extract, Ethyl acetate, and Butanol extract were obtained from fractionation of previously reported aqueous ethanolic extract of *T. cordifolia* and tested for neuroprotective activity. Out of the four fractions, Butanol extract of *T. cordifolia* (B-TCE) exhibited neuroprotective potential by preventing degeneration of neurons induced by glutamate. Expression of different neuronal, apoptotic, inflammatory, cell cycle regulatory and plasticity markers was studied by immunostaining and Western blotting. Neurite outgrowth and migration were also studied using primary explant cultures, wound scratch and gelatin zymogram assay.

**Results:**

At molecular level, B-TCE pretreatment of glutamate-treated cultures normalized the stress-induced downregulation in the expression of neuronal markers (MAP-2, GAP-43, NF200) and anti-apoptotic marker (Bcl-xL). Further, cells exposed to glutamate showed enhanced expression of inflammatory (NF-κB, AP-1) and senescence markers (HSP70, Mortalin) as well as the extent of mitochondrial damage. However, B-TCE pretreatment prevented this increase and inhibited glutamate-induced onset of inflammation, stress and mitochondrial membrane damage. Furthermore, B-TCE was observed to promote regeneration, migration and plasticity of cerebellar neurons, which was otherwise significantly inhibited by glutamate treatment.

**Conclusion:**

These results suggest that B-TCE may have neuroprotective and neuroregenerative potential against catastrophic consequences of glutamate-mediated excitotoxicity and could be a potential therapeutic candidate for neurodegenerative diseases.

**Electronic supplementary material:**

The online version of this article (10.1186/s12906-018-2330-6) contains supplementary material, which is available to authorized users.

## Background

The challenging diversity of neurological disorders such as trauma, ischemia, stroke, epilepsy as well as neurodegenerative diseases, although have different initial causes of disease onset but share a common final destructive pathway known as excitotoxicity [1]. L-glutamic acid is a major excitatory amino acid in the CNS, which plays a major role in neurotransmission and is responsible for performing fundamental brain functions such as neuronal circuit formations and synaptic plasticity underlying memory and cognition [2]. Glutamate acts through both inotropic as well as metabotropic receptors and increased extracellular levels of glutamate lead to overactivation of glutamate receptors resulting in neuronal damage [3]. Higher concentration of glutamate released during hypoxia or ischemia causes overstimulation of glutamate receptors and rise in intracellular calcium levels [4]. Higher intracellular calcium further activates various enzymes such as proteases, endonucleases, phospholipases and nitric oxide synthase (NOS), thus enhancing structural degradation, mitochondrial damage, ROS/RNS production, DNA damage and increased expression of inflammatory mediators which lead to increased neuronal damage [5, 6].

The currently available drug therapies for neurodegenerative diseases are palliative with limited effectiveness and adverse side effects [7–9]. The major challenge for the researchers is to develop a therapy that addresses the underlying cause/mechanism of degeneration with improved effectiveness and least side effects. Therapeutic interventions that modify the progression of neurodegeneration may prove useful and plant-based interventions offer various possibilities to modify the disease progression and symptoms [8]. *T. cordifolia*, a Rasayana herb of Indian Ayurvedic system has been reported to possess anti-cancer, anti-oxidative, anti-diabetic, anti-aphrodisiac, adaptogenic, immune stimulant and immune protective activities [10–14]. However, neuroprotective activity of this plant is least explored. Recently, the ethanolic extract of *T. cordifolia* was reported to exhibit neuroprotective activity against 6-OHDA induced Parkinsonism [15]. Recent studies from our lab have reported that 50% aqueous ethanolic extract of *T. cordifolia* (TCE) ameliorated anxiety, improved exploratory behavior and modulated synaptic plasticity in sleep-deprived rats [16]. Various medicinal properties of *T. cordifolia* have been attributed to its phytochemical constituents belonging to different classes such as alkaloids, terpenoids, glycosides, sesquiterpenoids, aliphatic compounds and steroids. Some alkaloids, glycosides and aliphatic compounds are broadly considered responsible for immune modulatory and neuroprotective properties of this herb [17–19]. n-Butanol fraction of *T. cordifolia* extract has been reported to have tinocordifolioside A and tinocordiside as active compounds [20, 21].

The current study was aimed to investigate the neuroprotective potential of Butanol extract of *T. cordifolia* (B-TCE) against glutamate-induced excitotoxicity using primary cerebellar neurons as a model system. Cerebellum constitutes the major neuronal population of the nervous system. A homogenous population of cerebellar granular neuronal cells developed postnatally from new born rats and mice has been widely accepted as a cellular model system to study various aspects of neurogenesis, neuronal development, death and other brain pathologies [22, 23]. Primary cerebellar neuronal cultures were established using 6-day old rat pups and were treated with glutamate, B-TCE alone and B-TCE + glutamate. After initial microscopic observations, we further investigated the interplay between glutamate and B-TCE on the expression of different molecular effectors responsible for neuronal structural integrity, senescence, apoptosis, inflammation and neuronal plasticity.

## Methods

### Preparation of plant extract

*T. cordifolia* was collected in last week of January, 2015 from the local forest in Ropar district of Punjab, India and was identified by Dr. Amarjeet Singh Soodan, Herbarium in-Charge of Department of Botanical and Environmental Sciences, Guru Nanak Dev University, Amritsar, India. A voucher specimen of stem and leaves has been deposited in departmental herbarium with reference no. 65 Bot. & Env. Sc. Dated 04-09-2017. Initially, 50% aqueous ethanolic extract was prepared by percolating 1.5 kg dry stem powder in a percolator with 5 L capacity for 4 times. Collected extract was evaporated at 45 °C using rotavapor and lyophilizer yielding 220 g of TCE which was further fractionated with n-Hexane, Chloroform, Ethyl acetate and n-Butanol (SRL, Analytical grade). Each fraction was collected and evaporated to dryness using a rotary evaporator which yielded 1.17 g Hexane extract, 10 g Chloroform extract, 12 g Ethyl acetate extract and 56.2 g Butanol extract (B-TCE). For use in culture, 100 mg/mL stock was prepared in DMSO and diluted in neurobasal medium (Invitrogen, CA, USA) to the final concentration of 20 μg/mL according to experimental requirements.

### Primary cerebellar neuronal and explant cultures

Primary cerebellar and neuronal cultures were obtained from 6-day old albino Wistar rat pups. Briefly, rat pups were sacrificed by decapitation and brain was removed out from the skull. Cerebellum was dissected out in chilled 1X PBS and after removal of meninges, it was transferred to Petri dish containing fresh chilled 1X PBS. Three cuts were given in a cerebellum and 1X PBS was replaced with 0.05% Trypsin-EDTA containing DNase I (2 units/mL) (Invitrogen). Trypsinization was carried out for 10 min at 37 °C with 2–3 intermediate gentle shakings, followed by addition of equal volume of neurobasal medium for stopping the digestion. Partially digested tissue was centrifuged for 2 min (1000 rpm) and the pellet was re-suspended in 4 mL chilled neurobasal medium. To obtain single cell population, the pellet was triturated with micropipette (25 strokes) and allowed to settle down for 5–10 min. Leaving the debris undisturbed, the suspension was then collected into a fresh tube and centrifuged for 2 min at 1000 rpm. Obtained pellet was re-suspended in 1 mL neurobasal medium (normalized to room temperature), counted using hemocytometer and seeded on Poly-L-Lysine (PLL) coated coverslips in 12 or 24 well plates according to experiment.

For explant culture, after removing meninges, cerebellum was chopped into very small pieces using scalpel or micro-dissector. These small pieces of the cerebellar tissue were placed onto PLL coated coverslips with the help of micropipette, allowed to attach for few minutes followed by neurobasal medium replenishment in the wells. At least three explants per well were established and the experiment was carried out in triplicates.

### Cell culture and treatments

Primary cerebellar neurons were seeded in 12 or 24 well plates containing PLL coated coverslips at a seeding density of 40,000 cells/mL. Four groups were studied 1) Control 2) Glutamate treatment 3) B-TCE alone treatment 4) B-TCE + Glutamate treatment. After 24 h of seeding, group 3 and 4 were treated with 20 μg/mL of B-TCE and group 1 and 2 were given medium change only. After the next 24 h, glutamate was added to group 2 and 4 at a final concentration of 2 mM and incubation was done for another 24 h. Control cultures i.e. group 1 was given only medium change. Cultures were maintained in neurobasal medium containing B27, bFGF supplement (Invitrogen) and were incubated in a humidified 5% CO_2_ incubator at 37 °C temperature. From the reported literature, we initially checked 1, 2 and 5 mM concentration of glutamate on primary cerebellar neurons and selected 2 mM concentration as a subtoxic dose [31]. Two different concentrations of B-TCE (10 and 20 μg/mL) were tested against 2 mM glutamate out of which 20 μg/mL was more effective, so it was selected for further experiments (Additional file 1: Fig. S1). Each experiment was carried out in triplicate.

### Cellular and nuclear morphological studies

After completion of treatment regime i.e. 72 h of seeding, primary cerebellar neurons were observed and phase contrast images were captured using EVOS FL microscope (Invitrogen). Further, to gain detailed information about the effect of glutamate and B-TCE pretreatment on number of processes or length of processes, morphometric study was carried out. Cells were seeded at a seeding density of 20,000 cells/mL in PLL coated 12 well plates, followed by the treatment regime mentioned above and harvested by fixing in 2.5% glutaraldehyde (in neurobasal medium). After fixing, cells were stained with staining solution containing 1% methylene blue and 1% toluidine blue in 1% sodium tetraborate for 40 min, followed by rinsing with distilled water and then allowed to dry. Images were captured using EVOS FL microscope and analyzed with Image Pro Plus software from media cybernetics version 4.5.1. The experiment was performed in triplicates and 100 cells from each group were analyzed for the study of number and length of processes. For nuclear morphology, cells were stained with a fluorescent stain 4′, 6-diamidino-2-phenylindole (DAPI) (Sigma-Aldrich, MO, USA) which specifically binds to AT-rich regions in DNA.

### Immunostaining

Control and treated cells were given a washing with chilled 1X PBS followed by fixation with acetone and methanol (1:1) and permeabilization with 0.3% Triton- X100 in 1X PBS. Cells were then blocked with 2% BSA and incubated with primary mouse monoclonal antibody anti-α-Tubulin (1:500), anti-NF-κB (1:500), anti-MAP-2 (1:250), anti-NF200 (1:500), anti-GAP 43 (1:250), anti-HSP70 (1:500), anti-Mortalin (1:500), anti-Bcl-xL (1:200), anti-Cyclin D1(1:250), anti-NCAM (1:250), rabbit monoclonal anti-AP-1(1:250) (all from Sigma-Aldrich) and mouse monoclonal anti-PCNA (1:250), mouse polyclonal anti-PSA-NCAM (1:250) (from Millipore, MA, USA) for 24 h in humid chamber at 4 °C. No permeabilization was carried out for PSA-NCAM immunostaining. After primary antibody incubation, three washings were given with 0.1% PBST and incubated with secondary antibody (goat anti-mouse/ rabbit IgG/ IgM Alexa Fluor 488/543) for 2 h at RT. Cells were stained with nuclear staining dye DAPI (Sigma-Aldrich) for 15 min, washed with 0.1% PBST and mounted with antifading agent Fluoromount (Sigma-Aldrich). Images were captured with Nikon AIR Confocal Laser Scanning Microscope and analyzed with NIS elements analysis software version 4.11.00 (Nikon Co., Tokyo, Japan). Each experiment was carried out in triplicate.

### Western blotting

For total protein extraction, Primary cerebellar neurons were grown and treated in multi-well plates followed by harvesting using chilled PBS–EDTA (1 mM). The cell pellet was homogenized in RIPA buffer (50 mM Tris (pH 7.5), 150 mM NaCl, 0.5% sodium deoxycholate, 0.1% SDS, 1.0% NP-40). Protein concentration was determined by the Bradford method and protein lysate (25 μg) was resolved in 7%, 10% and 12% gels by Sodium dodecyl sulfate- Polyacrylamide gel electrophoresis (SDS-PAGE), followed by semi-dry transfer onto a PVDF membrane (Hybond-P). Further, membranes were incubated with mouse monoclonal anti-α-Tubulin (1:5000), anti-NF-κB (1:5000), anti-MAP-2 (1:2500), anti-NF200 (1:5000), anti-GAP-43 (1:2500), anti-HSP70 (1:5000), anti-Mortalin (1:5000), anti-Bcl-xL (1:2000), anti-Cyclin D1(1:2500), anti-NCAM (1:2500), mouse polyclonal anti-PSA-NCAM (1:2500), and rabbit monoclonal anti-AP-1(1:2500) antibodies for overnight at 4 °C. This was followed by washing with 0.1% TBST and incubation with HRP labeled secondary antibodies for 2 h at RT. Immunoreactive bands were detected by ECL Plus Western blot detection system using LAS 4000 (Amersham Biosciences, GE Healthcare, UK). The final expression of each protein of interest was calculated after normalizing the protein expression with expression of endogenous control α-tubulin in the same sample. The change in expression of the gene of interest was calculated from average of IDV (integrated density values) obtained from at least three independent experiments.

### mRNA expression

Total RNA was extracted from the cells by TRI reagent (Sigma-Aldrich) according to manufacturer’s instructions and cDNA was synthesized from it. 50 ng of cDNA was used for reaction in 5 μL of reaction mixture in triplicate containing 2.5 μL of 2X TaqMan Master Mix, 0.5 μL of 20X predesigned Primer Probe mix (Applied Biosystem, CA, USA) and 1 μL of water, using amplification Step One Plus Real-Time PCR system (Applied Biosystem). Amplification conditions comprised of initial holding stage of 50 °C for 2 min followed by 95 °C for 10 min, and then cycling stage comprised of 40 cycles of amplification (denaturation at 95°C for 15 sec, further annealing and elongation at 60 °C for 1 min). GAPDH was used as an endogenous control for each gene of interest. The relative gene expression of each candidate gene was calculated by ‘Livak method’ and represented as 2^−ΔΔCt^ and final gene expression as 2^−ΔΔCt^±SEM. Final results were obtained as average of minimum three different observations for each experimental group.

### Pro-inflammatory cytokine ELISA based determination

Media was collected from different wells of all the four different treatment groups and used for determination of pro-inflammatory cytokines using sandwich ELISA based kits from Cayman Chemical Company, USA (TNF-α and IL-6) and Sigma Aldrich, USA (IL-1β). Estimation and calculations were performed as per manufacturer’s protocol. The experiment was performed at least three times in triplicate.

### Wound scratch assay

To study the effect of B-TCE on migration behavior of primary neurons, primary cerebellar neurons were seeded at a high density and grown to achieve confluency. A straight scratch was given with microtip on all the coverslips containing a confluent monolayer of cells which was followed by treatment with Glutamate (2 mM), B-TCE (20 μg/mL), B-TCE (20 μg/mL) + Glutamate (2 mM). Phase contrast images were captured at zero and 24 h of treatment using EVOS FL microscope. Gap closure was calculated after image analysis by Image-Pro Plus software version 4.5.1 from the media cybernetics. The distance cells migrated into the cell-free area was measured with respect to the initial cell-free area to determine percent gap closure.

### Mitotracker staining

To carry out Mitotracker staining, after completion of treatment regime, Mitotracker green FM (Invitrogen) was added to all the treatment groups in multi-well plate at 100 nM final concentration and incubated in CO_2_ Incubator at 37 °C. After 45 min of incubation, the medium was discarded, cells were washed with chilled 1X PBS and fixed using chilled acetone and methanol (1:1) solution. Coverslips containing cultures were mounted using antifading medium and captured using Nikon A1R confocal microscope on the same day.

### Gelatin zymogram study

In order to study the effect of glutamate and B-TCE on Matrix Metalloproteinases expression, cell culture medium was collected from different treatment groups, briefly spun to remove any floating debris and supernatant samples were separated on 10% SDS–polyacrylamide gels containing 0.1% gelatin. Further, gels were incubated in renaturation buffer (Invitrogen) for 1 h followed by 3 washings with distilled water. Gels were then incubated in developing buffer (Invitrogen) for 72 h at RT on a platform rocker. After 72 h, gels were washed again in distilled water, stained with Coomassie Brilliant Blue (CBB) and destained using buffer containing 10% acetic acid and 50% methanol (*v*/v). Clear white bands in blue stained gel were considered as regions of gelatinolytic activity.

### UPLC/MS analysis of B-TCE

In order to determine different compounds present in B-TCE, it was subjected to LC/MS profiling. 10 mg B-TCE was dissolved in 1 mL methanol, vortexed and passed through 0.22 μm filter. 1 μL sample was subjected to Waters Acquity UPLC system (Waters, MA, USA) with Acquity UPLC BEH C_18_ column (100 mm × 2.1 mm, particle size 1.7 μm) and photodiode array (PDA) detector. The sample was separated using mobile phase solvent gradient consisting of 1% formic acid in water (A) and 1% formic acid in acetonitrile (B). MS was performed using Q-TOF triple Quadrupole Mass Spectrometer attached with Electrospray Ionisation (ESI) source (Waters Micromass, Manchester, UK). LC/MS analysis was performed using Masslynx v4.1.

### Statistical analysis

Values are expressed as mean ± SEM from at least three independent experiments. Results were analyzed to determine the significance of means by using one way ANOVA (Holm-Sidak post hoc method), which was performed by Sigma Stat software (Version 3.5) for Windows. Values with *p* ≤ 0.01 were considered as statistically significant. Data were compared between control and other groups such as glutamate, B-TCE and B-TCE + glutamate (^*^p ≤ 0.01) as well as glutamate alone with B-TCE and B-TCE + glutamate groups (^#^*p* ≤ 0.01).

## Results

### B-TCE modulated the effect of glutamate on cellular and nuclear morphology

**Fig. 1:**
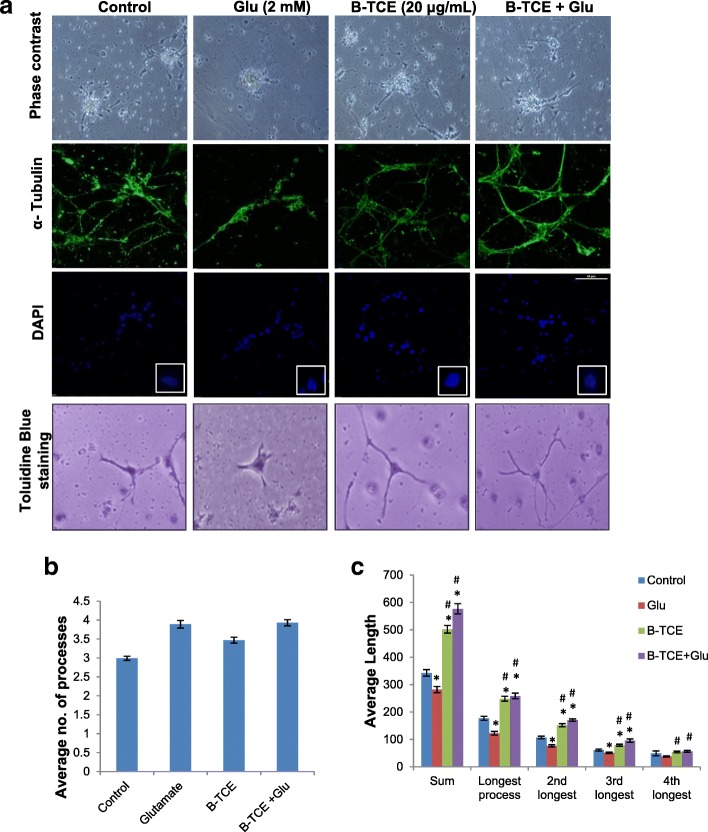
B-TCE pretreatment inhibited adverse effects of glutamate on cellular and nuclear morphology. **a**) Phase contrast, α-Tubulin immunostained and nuclear staining (DAPI) confocal micrographs and Toluidine blue stained Control, Glutamate (2 mM), B-TCE (20 μg/mL) and B-TCE + Glu treated primary cerebellar neurons. **b**) The histogram represents average number of processes and **c**) average length of processes of primary cerebellar neurons of these four different groups. Data was compared between Control and other groups such as Glutamate, B-TCE and B-TCE + Glu (^*^p ≤ 0.01) as well as Glutamate alone with B-TCE and B-TCE + Glu groups (^#^*p* ≤ 0.01)

Effect of glutamate and B-TCE pretreatment was initially studied by phase contrast microscopy followed by confocal imaging for α- tubulin immunostaining. Glutamate exposure was observed to degenerate primary cerebellar neurons, however, B-TCE pretreated culture exhibited healthy morphology with long and stellate processes as seen in control and B-TCE alone treatment groups (Fig. 1a). B-TCE alone treatment promoted defasciculation (Fig. 1a). To study the effect of glutamate and B-TCE on number and length of processes of primary cerebellar neurons, morphometric analysis from toluidine and methylene blue stained cells was carried out. It was observed that number of processes in all the three treatment groups i.e. glutamate, B-TCE alone and B-TCE pretreatment followed by glutamate were although significantly higher than control (*p* ≤ 0.01) (Fig. 1b), but the sum length as well as length of individual processes were significantly reduced by glutamate (Fig. 1c). However, B-TCE pretreatment was seen to increase the length of processes significantly (*p* ≤ 0.01) (Fig. 1a and c). Further, to observe nuclear condensation, which is considered as induction of apoptosis, nuclei were stained with DAPI. Number of apoptotic cells was calculated in each group and 67% of total population was found to be apoptotic in glutamate-treated group, whereas, only 19.5% and 15.9% cells showed condensed nuclear morphology in control and B-TCE alone group, respectively. B-TCE pretreatment significantly reduced apoptotic cell population to 23.9% (*p* ≤ 0.01), thus suggesting that B-TCE suppressed the onset of apoptosis (Fig. 1a and Additional file 1: Fig. S2). As the cultures treated with B-TCE alone showed cellular and nuclear morphology similar to control, it may be suggested that B-TCE did not have any adverse effects on primary cerebellar neurons.

### B-TCE suppressed glutamate-induced neuronal degeneration by supporting neuronal differentiation and maturation

**Fig. 2:**
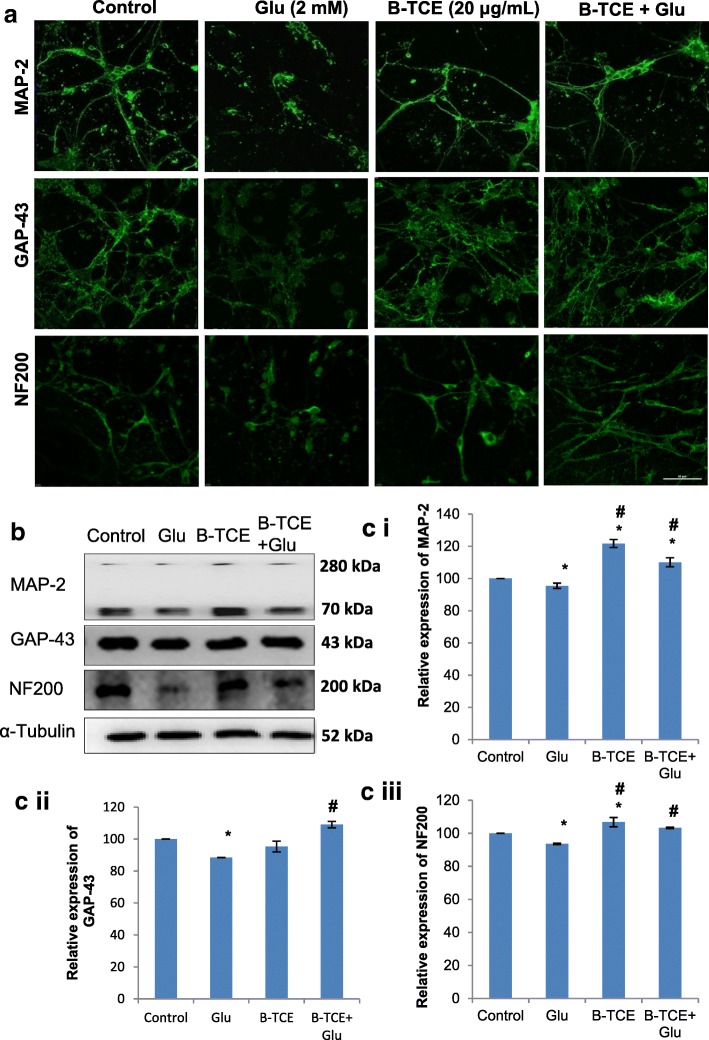
B-TCE pretreatment suppressed the neuronal degeneration induced by glutamate by modulating the expression of neuronal structural markers. **a**) Confocal micrographs of Control, Glutamate (2 mM), B-TCE (20 μg/mL) and B-TCE + Glu treated primary cerebellar neurons immunostained for MAP-2, GAP-43 and NF200. **b**) Representative immunoblots for MAP-2, GAP-43 and NF200 where α-Tubulin was used as internal control. **c**) Histogram representing relative expression of MAP-2 (i), GAP-43 (ii) and NF200 (iii) obtained from normalized relative optical densities of bands. Data was compared between Control and other groups such as Glutamate, B-TCE and B-TCE + Glu (^*^p ≤ 0.01) as well as Glutamate alone with B-TCE and B-TCE + Glu groups (^#^*p* ≤ 0.01). Confocal Images were captured at 60X objective (Scale bar: 50 μm)

To study the molecular basis of glutamate-induced neurodegeneration, immunostaining for neuronal markers MAP-2, GAP-43 and NF200 was carried out in different groups. MAP-2 and NF200 are neuron specific proteins which are responsible for neuronal spindle formation and axonal caliber in dendrites of post-mitotic neurons [24, 25], whereas, GAP-43 has been reported to express at neuronal growth cones during development and axonal regeneration. The decrease was observed in expression of all the three markers after glutamate exposure, but their expression was found comparable to the control group in B-TCE pretreatment and B-TCE alone treatment groups in immunostaining (Fig. 2a). This immunostaining data was further supported by Western blotting results, where glutamate treated group showed a significant decrease in MAP-2, GAP-43 and NF200 expression as compared to control group (*p* ≤ 0.01). B-TCE pretreatment significantly normalized the glutamate-induced reduction in expression of these markers (*p* ≤ 0.01). B-TCE alone treatment was found to increase MAP-2 and NF200 significantly w.r.t control. MAP-2 has three isoforms MAP-2a, MAP-2b and MAP-2c (280 kDa, 280 kDa and 70 kDa, respectively) out of which MAP-2b and 2c have been reported to be present in new born rat pups brain. In the given blot, these two isoforms were detected by monoclonal mouse anti-MAP-2 antibody (Cat# M9942, Sigma-Aldrich). The histogram for MAP-2 represents combined analysis for both the isoforms (Fig. 2b). These observations suggest that B-TCE pretreatment suppresses glutamate-induced reduction of neuronal protein expression.

### B-TCE abolished the onset of inflammation as a result of glutamate-induced excitotoxicity

**Fig. 3:**
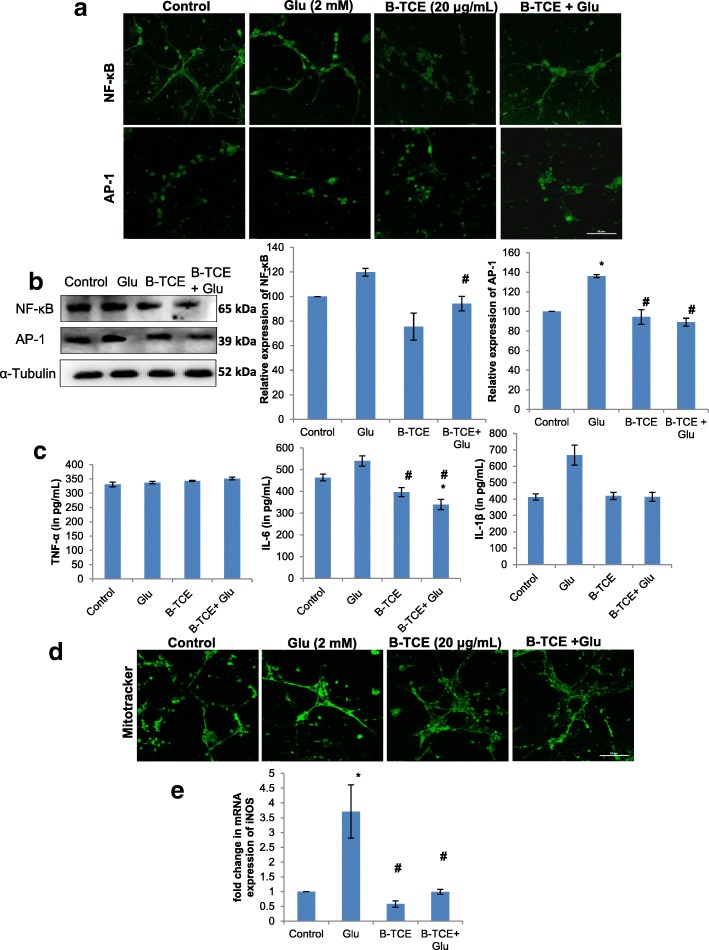
B-TCE pretreatment abolished the glutamate-induced onset of inflammation. **a**) Confocal micrographs of Control, Glutamate (2 mM), B-TCE (20 μg/mL) and B-TCE + Glu treated primary cerebellar neurons immunostained for NF-κB and AP-1. **b**) Representative immunoblots and histograms showing relative expression of NF-κB and AP-1 where α-Tubulin was used as internal control. **c**) Histograms representing secretory levels of pro-inflammatory cytokines TNF-α, IL-6 and IL-1β (in pg/mL) in the four different treatment groups. **d**) Confocal micrographs of primary cerebellar neurons stained with Mitotracker green FM **e**) Histogram representing fold change in mRNA expression of iNOS in four different treatment groups. Data was compared between Control and other groups such as Glutamate, B-TCE and B-TCE + Glu (^*^p ≤ 0.01) as well as Glutamate alone with B-TCE and B-TCE + Glu groups (^#^*p* ≤ 0.01). Confocal Images were captured at 60X objective (Scale bar: 50 μm)

To further elucidate whether B-TCE pretreatment suppresses glutamate-induced inflammation, expression of inflammatory markers NF-κB and transcription factor AP-1 was studied by immunostaining and Western blotting. A significant increase in NF-κB and AP-1 expression was observed in glutamate-treated cells, whereas, B-TCE pretreatment significantly reduced the expression of these markers as compared to glutamate alone group (*p* ≤ 0.01). The immunostaining data was supported by Western blotting results (Fig. 3a and b). Secretory levels of pro-inflammatory cytokines TNF-α, IL-6 and IL-1β in culture media were assayed by ELISA. Although no significant change was observed in levels of TNF-α but a pronounced increase was found in IL-6 and IL-1β levels in primary cerebellar neurons exposed to glutamate, which was significantly suppressed in B-TCE pretreated culture (*p* ≤ 0.01) (Fig. 3c). Mitochondrial dysfunction and increased oxidative stress are the well-known consequences of glutamate-induced excitotoxicity. Further, to test whether B-TCE pretreatment attenuates the mitochondrial membrane damage and oxidative stress induced by glutamate, Mitotracker green FM staining and iNOS mRNA expression were studied. Change in intensity of mitotracker staining is related to altered mitochondrial activity. Significantly higher intensity of mitotracker was observed in glutamate-treated primary cerebellar neurons indicating higher oxidative stress and membrane damage, which was prevented by B-TCE pretreatment as indicated by intensity of mitotracker staining comparable to control group (Fig. 3d). Glutamate-induced rise in iNOS expression (3.7 fold) at transcriptional level was significantly inhibited by B-TCE pretreatment (*p* ≤ 0.01) (Fig. 3e). These observations suggested that B-TCE pretreatment abolished glutamate-induced rise in inflammatory protein expression, secretory levels of pro-inflammatory cytokines, mitochondrial membrane damage and iNOS expression.

### B-TCE normalized glutamate-induced enhanced expression of stress chaperone proteins

**Fig. 4:**
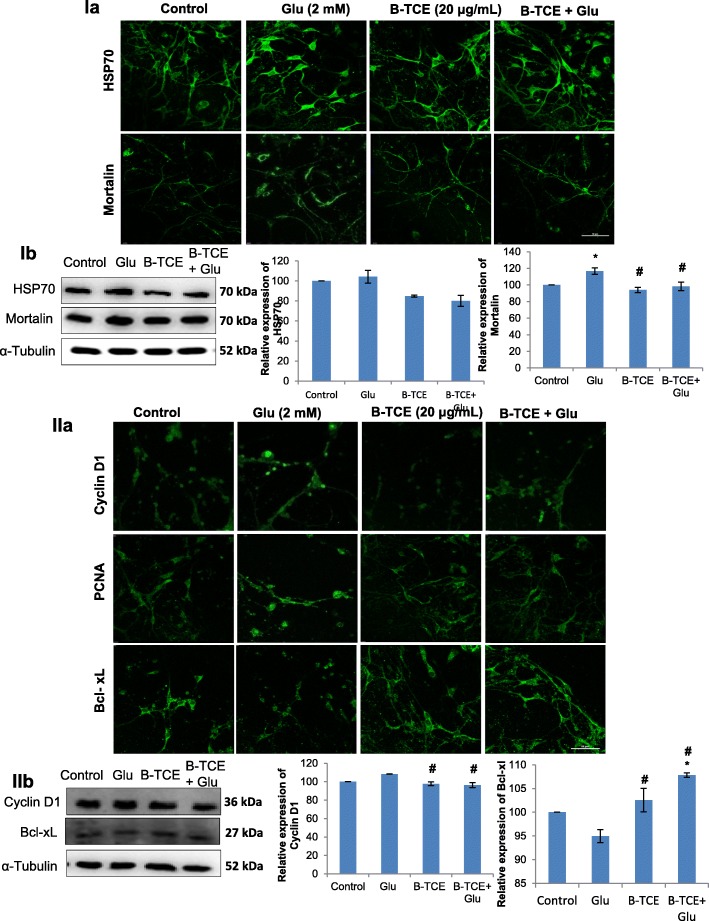
**I**) B-TCE pretreatment normalized glutamate-induced increase in stress chaperones expression. **a**) Confocal micrographs of Control, Glutamate (2 mM), B-TCE (20 μg/mL) and B-TCE + Glu treated primary cerebellar neurons immunostained for HSP70 and Mortalin. **b**) Representative immunoblots and histograms showing relative expression of HSP70 and Mortalin. **II**) B-TCE pretreatment regulated the expression of cell cycle and pro-apoptotic proteins during glutamate exposure. **a**) Confocal micrographs of Control, Glutamate (2 mM), B-TCE (20 μg/mL) and B-TCE + Glu treated primary cerebellar neurons immunostained for Cyclin D1, PCNA and Bcl-xL. **b**) Representative immunoblots and histograms showing relative expression of Cyclin D1 and Bcl-xL where α-Tubulin was used as internal control. Data was compared between Control and other groups such as Glutamate, B-TCE and B-TCE + Glu (^*^p ≤ 0.01) as well as Glutamate alone with B-TCE and B-TCE + Glu groups (^#^*p* ≤ 0.01). Confocal Images were captured at 60X objective (Scale bar: 50 μm)

To investigate the effect of glutamate on stress chaperone proteins expression and whether B-TCE abrogates these changes, expression of stress chaperones, i.e. heat shock protein HSP70 and Mortalin was studied. HSP70 and Mortalin protect neurons from protein aggregation and toxicity. Immunostaining data revealed increase in expression of HSP70 and Mortalin in glutamate exposed group, whereas, B-TCE pretreatment suppressed this increase (Fig. 4 Ia). The Western blot data also showed that glutamate treatment enhanced the expression of HSP70, however, the change was not statistically significant (Fig. 4 Ib). B-TCE pretreatment reduced the expression of HSP70 as compared to both control and glutamate treated group. Further, glutamate treated cultures showed significant increase in Mortalin expression (*p* ≤ 0.01) as compared to control as well as B-TCE pretreatment group (Fig. 4 Ib). These results suggest that B-TCE pretreatment protected cells from glutamate-induced increase in stress proteins (Fig. 4 Ia and Ib).

### B-TCE regulated glutamate-induced changes in cell cycle proteins and apoptotic marker

Abnormal cell cycle regulation has been reported to be associated with neurodegeneration [26]. To further explore whether B-TCE modulates expression of cell cycle regulators, Cyclin D1 and PCNA expression was studied. Enhanced expression of Cyclin D1 and PCNA was observed in glutamate exposed primary cerebellar neurons. However, B-TCE pretreatment significantly reduced the Cyclin D1 and PCNA expression to near control levels (*p* ≤ 0.01) (Fig. 4 IIa). Western blotting for Cyclin D1 also supported these observations (Fig. 4 IIb). Further, we studied the expression of anti-apoptotic marker Bcl-xL which showed significant downregulation in glutamate-treated culture (*p* ≤ 0.01). On the other hand, significantly higher expression of Bcl-xL was observed in B-TCE alone and B-TCE pretreatment followed by glutamate treatment as compared to glutamate treated group (*p* ≤ 0.01) (Fig. 4 IIa and IIb). These observations may suggest that glutamate-induced excitotoxicity induced aberrant cell cycle progression of primary cerebellar neurons in cell cycle, reduced the levels of anti-apoptotic protein Bcl-xL which may result in induction of apoptosis, whereas, B-TCE pretreatment inhibited cell cycle reactivation and maintained normal levels of Bcl-xL, thus inhibiting apoptosis induction.

### B-TCE promoted plasticity, neurite outgrowth and migration

**Fig. 5:**
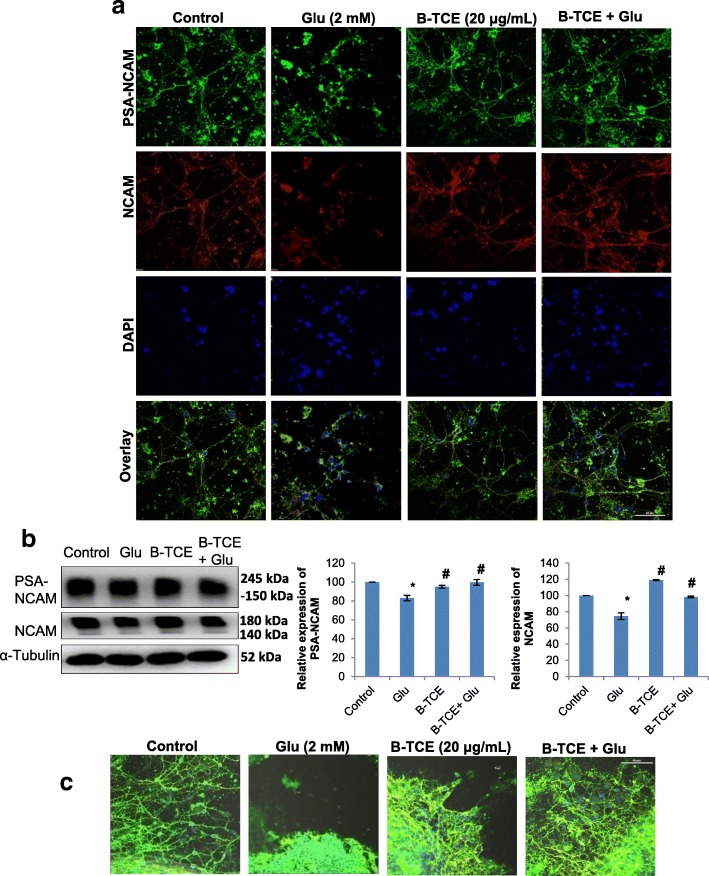
B-TCE pretreatment promoted plasticity, neurite outgrowth and migration and protected from glutamate-induced excitotoxicity. **a**) Confocal micrographs of Control, Glutamate (2 mM), B-TCE (20 μg/mL) and B-TCE + Glu treated primary cerebellar neurons immunostained for PSA-NCAM and NCAM. Nuclei were stained with DAPI (third panel). The fourth panel represents the overlay of above three panels. **b**) Representative immunoblots and histograms representing relative expression PSA-NCAM and NCAM where α-Tubulin was used as internal control. **c**) Overlay confocal micrographs of primary cerebellar explants stained for PSA-NCAM, NCAM and DAPI. Data was compared between Control and other groups such as Glutamate, B-TCE and B-TCE + Glu (^*^p ≤ 0.01) as well as Glutamate alone with B-TCE and B-TCE + Glu groups (^#^*p* ≤ 0.01). Confocal Images were captured at 60X objective (Scale bar: 50 μm)

**Fig. 6:**
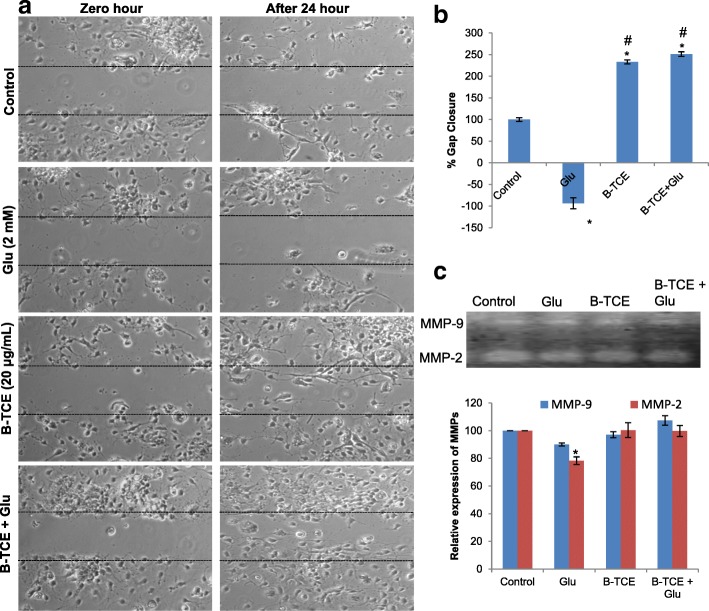
B-TCE pretreatment promoted migration into the scratched area under glutamate-induced excitotoxic insult. **a**) Phase contrast micrographs of Control, Glutamate (2 mM), B-TCE (20 μg/mL) and B-TCE + Glu treated primary cerebellar neurons captured at 0 and 24 h of treatment. **b**) Representative histogram showing percentage gap closure after 24 h of treatment **c**) Gelatin zymogram showing MMP bands and histogram representing densitometric analysis of corresponding MMP bands. Data was compared between Control and other groups such as Glutamate, B-TCE and B-TCE + Glu (^*^p ≤ 0.01) as well as Glutamate alone with B-TCE and B-TCE + Glu groups (^#^*p* ≤ 0.01). Phase contrast Images were captured at 20X objective

Glutamate neurotransmission plays an important role in synaptic plasticity, however, abnormal levels of glutamate pose detrimental effects on plasticity development and regulation [27]. The effect of glutamate treatment was also studied on plasticity markers PSA-NCAM and cell adhesion molecule NCAM in primary cerebellar neurons. Glutamate exposure significantly reduced PSA-NCAM and NCAM expression as compared to control cells, whereas, B-TCE pretreated cultures showed expression of these proteins comparable to control group (*p* ≤ 0.01). However, a significant difference in expression was observed as compared to glutamate alone treated group (*p* ≤ 0.01) (Fig. 5a and b). Immunostaining and Western blotting data are in line with each other. NCAM blot shown two bands of 180 kDa and 140 kDa as due to alternative splicing during RNA processing, three isoforms of NCAM 180 kDa, 140 kDa and 120 kDa exist. The monoclonal anti-NCAM antibody, clone NCAM OB11 (Cat# 9672, Sigma-Aldrich) used for Western blotting detects 180 kDa and 140 kDa bands, justifying the two bands observed in Western blot in Fig. 5b. Further, to study the effect of glutamate on neurite outgrowth and migration, primary cerebellar explant cultures were established. There was no outgrowth observed from glutamate treated explants which also showed low expression of NCAM and PSA-NCAM. However, visible cells with long processes were observed to be migrating from B-TCE pretreated explants with enhanced expression of NCAM and PSA-NCAM (Fig. 5c). To gain more insight into the effect of glutamate exposure and B-TCE treatment, wound scratch assay and Gelatin zymography were performed. A widened gap with negative gap closure (− 94%) was observed in primary cerebellar neuronal cultures treated with glutamate as compared to control cultures (gap closure taken as 100%), whereas, B-TCE and B-TCE + Glutamate treated cells showed almost double percent gap closure (233% and 251%, respectively) (Fig. 6a and b). Furthermore, significantly reduced expression of MMP-2 was also observed in glutamate-treated cells as compared to control, whereas, no change was observed in other two groups (*p* ≤ 0.01) (Fig. 6c). These observations suggest that glutamate exposure to cellular explants reduced neuronal plasticity, inhibited neurite outgrowth and migration of primary cerebellar neurons, whereas, B-TCE pre-treatment rescued the primary cerebellar neurons from these adverse effects of glutamate excitotoxicity, maintained plasticity and promoted neurite outgrowth and migration.

### Phytochemical characterization of B-TCE by UPLC-MS

*T. cordifolia* has been reported to consist of a variety of phytochemicals such as alkaloids, glycosides, diterpenoid lactones, steroids, aliphatic compounds and others [17, 28]. Since B-TCE was a butanolic fraction of crude extract of *T. cordifolia* and was observed to show neuroprotective activity, so to study the active compounds UPLC-MS of B-TCE was carried out. Peaks of alkaloids and glycosides were identified on the basis of reported literature for *T. cordifolia*. Peaks of magnoflorine, palmatine, norcoclaurine, cordifolioside A, oblongine, tetrahydropalmatine, 11-hydroxy mustakone and tinocoridiside were identified on the basis of reported mass and mass fragments described in the literature of phytochemical characterization of *T. cordifolia* (Table 1) [29, 30]. [Chromatograms at different wavelengths, TIC and spectra corresponding to retention time are included in supplementary material and identified mass peaks and mass fragments denoted by *(Additional file 1: Fig. S3-S12)].

**Table 1:** UPLC-MS analysis: Observed molecular weight and mass fragments of identified compounds in B-TCE extract

S. No.	Compound	RT	m/z	MS2	Nature	Reference
1.	Magnoflorine	1.135	343.14 (M + H)^+^	327, 296	Alkaloid	[29, 30]
2.	Palmatine	3.607	353 (M + H)^+^	337, 308	Alkaloid	[29]
3.	Norcoclaurine	3.701	272 (M + H)^+^	255, 237	Alkaloid	[30]
4.	Cordifolioside A	3.756	527.25 (M + Na)^+^	210, 193	Glycoside	[29]
5.	Oblongine	5.597	314 (M)^+^	269,175	Alkaloid	[30]
6.	Tetrahydropalmatine	6.620	356.18 (M + H)^+^	340, 164	Alkaloid	[30]
7.	11-Hydroxy mustakone	6.973	235.21 (M + H)^+^	217, 161, 135	Sesqui-terpenoid	[30]
8.	Tinocordiside	6.973	419.25 (M + Na)^+^	217, 235	Glycoside	[29]

## Discussion

Glutamate-mediated excitotoxicity is the common final destructive pathway in the majority of neurodegenerative diseases and therapeutic strategies inhibiting or providing protection against excitotoxicity induced degeneration are much in the interest of researchers. The current study was aimed to study the neuroprotective potential of Butanol extract of *T. cordifolia* against glutamate-induced excitotoxicity. Initially, our lab has reported anti-proliferative and differentiation-inducing potential of 50% aqueous ethanolic extract of *T. cordifolia*. In an attempt to dissect out the active principle and to find effective lower dose we fractionated TCE with solvents of lower to higher polarity i.e. chloroform, hexane, ethyl acetate and butanol. The Chloroform and Hexane fractions (Chl-TCE and Hex-TCE) were found to exhibit anti-cancer activity against U87MG and IMR-32 cancerous cell lines at a very low dose as compared to TCE. Ethyl acetate fraction exhibited no specific effect on these cell lines, whereas, Butanol fraction exhibited neuroprotective potential. Different doses of Butanol fraction i.e. B-TCE were studied on primary cultures in combination with excitotoxic doses of glutamate as reported in the literature and changes in cell viability and morphology were observed. 2 mM concentration of glutamate was selected as toxic concentration against which 20 μg/mL B-TCE was found to exhibit protection. So, 2 mM glutamate and 20 μg/mL of B-TCE were selected for all the experiments. Generally, cerebellar granular cells are characterized by their long processes with defasciculated morphology, which were observed to undergo degeneration under neurotoxic insults.

Increased Ca^2+^ levels due to glutamate excitotoxicity have been reported to induce activation of catabolic enzymes, which causes degradation of majority of neuronal structural proteins including α-Tubulin, neurofilament peptides and microtubule-associated proteins [5]. In the current study, glutamate exposure to primary cerebellar neurons induced structural degradation as was evident from phase contrast micrographs, confocal images of α-Tubulin immunostaining and morphometric studies (Fig. 1). Fasciculated morphology and significantly reduced average process length (*p* ≤ 0.01) of glutamate-treated primary cerebellar neurons are supported by previously reported reduced dendritic branching and retraction of processes in the presence of toxic concentrations of excitotoxic stimuli [32]. In addition to changes in cellular processes, higher population with nuclear condensation (67%) was also observed which indicates induction of apoptosis by glutamate treatment. However, pretreatment of cerebellar neurons with B-TCE before glutamate exposure suppressed these adverse effects by maintaining structural and nuclear integrity as evident from reduced apoptotic cell population (23.9%) as well as increase in process length. B-TCE alone treatment also promoted defasciculation which allows better axonal branching. Further, toxicity in the neuronal environment has been reported to regulate the expression of neuronal markers [33]. MAP-2 and NF200 are structural proteins of mature neurons which characteristically express in dendrites, perikaryon and axons of post-mitotic neurons [25, 34]. Both of these proteins were downregulated by glutamate treatment. GAP-43, the other neuronal growth and plasticity protein which is expressed in growth cones of developing neurons was also found to be downregulated by glutamate exposure [32, 35]. B-TCE pretreated groups showed higher expression of these structural proteins as compared to glutamate treated group. The data may suggest that B-TCE exhibited neuroplastic and neuroprotective response by suppressing the glutamate-induced decrease in MAP-2, GAP-43 and NF200 expression in primary cerebellar neurons. Glutamate treatment has been reported to activate Calpain I (by elevating intracellular Ca^2+^ levels) which in turn downregulated the expression of structural proteins such as MAP-2 and NF200 in primary cortical, motor and hippocampal neurons. These findings were further confirmed by using Calpain I inhibitors which were found to ameliorate alterations in these structural proteins [36–38]. Calpains have also been reported to be involved in proteolysis of GAP-43 [39]. Although we have not studied Calpain I expression in the present work, but based on these literature reports on the glutamate-induced excitotoxicity and its effect on Calpain I activity, it may be suggested that B-TCE suppressed the changes in structural proteins by inhibiting activation of proteases like Calpain I.

Excitotoxic concentrations of glutamate result in oxidative stress and production of inflammatory mediators. Transcription factors NF-κB and AP-1 get activated with the onset of inflammation or stress, get translocated to the nucleus and induce transcription of pro-apoptotic and anti-apoptotic genes depending upon the stimuli. Rel A (P65) subunit of NF-κB has been recently reported to be activated by toxic concentrations of glutamate which further facilitates transcription of pro-apoptotic genes [40]. Pro-apoptotic genes’ transcription is also activated by AP-1 under glutamate excitotoxicity [41]. So, the increased levels of NF-κB and AP-1 in glutamate-treated group can be correlated with the onset of inflammation and induction of apoptosis, whereas, B-TCE pretreatment inhibited glutamate-induced activation of these transcription factors. Pro-inflammatory cytokines TNF-α, IL-6 and IL-1β secretion has been reported to increase during glutamate-induced excitotoxicity along with activation of apoptotic p38-MAPK protein [42]. Our data showed enhanced levels of IL-1β and IL-6, but not of TNF-α in glutamate-treated group, thus suggesting that NF-κB may be getting activated through receptors other than TNFR. Enhanced secretion of pro-inflammatory cytokines was also associated with increase in mRNA expression of an inducible form of nitric oxide synthase (iNOS), which may cause increase in synthesis of NO, which plays a major role in glutamate-induced oxidative stress and damage [43]. Further, the mitochondrial membrane was also found to be damaged after glutamate treatment. On the other hand, B-TCE pretreatment prevented the rise in pro-inflammatory cytokines, downregulated iNOS expression and mitochondrial membrane damage as is evident from the current data. These observations collectively suggest that B-TCE pretreatment abolished glutamate-induced onset of inflammation thus inhibiting apoptosis induction.

Neurodegenerative disorders are also known as proteinopathy disorders which involve aggregation and misfolding of proteins [44]. Heat shock proteins (HSP) are induced in response to various injuries such as stroke, trauma, neurodegenerative diseases and epilepsy and act in unison to repair or degrade the aggregated and misfolded proteins [44, 45]. In a previous report from our lab, HSP70, a central component of heat shock proteins was found to increase after glutamate-induced excitotoxicity in retinoic acid treated C6 glioma cells [46]. Upregulation in HSP70 expression has also been reported from animal models of focal ischemia and lithium-induced toxicity [47]. Mortalin, the other member of heat shock protein family is induced by glucose deprivation, metabolic stress, ionophores, ionizing radiation and toxins. Its concentration increases under oxidative stress as it is responsible for cellular homeostasis and tries combating with stress [48]. Under normal conditions, heat shock proteins play anti-apoptotic role, under neurotoxic insults they act as neuroprotective agent and under undealt oxidative stress, Mortalin triggers apoptosis by allowing cytoplasmic p53 activation [48]. These literature reports support upregulated HSP70 and Mortalin expression in glutamate-treated culture in the present study and may suggest that B-TCE pretreatment prevented misfolding of proteins, thus, abrogated the upregulation of expression of these stress chaperones by glutamate treatment.

Several cell culture and human post-mortem tissue studies have suggested the interconnection between activation of cell cycle and neurodegeneration [49]. Differentiated neurons attempt cell cycle re-entry and reactivation under various stress conditions such as nutrient deprivation, CNS injury and oxidative stress [26]. Oxidative stress and excitotoxic stimuli have been reported to induce DNA damage followed by the induction of DNA repair and synthesis, which results in inappropriate cell cycle entry leading to apoptosis and cell death [50]. Glutamate-induced increase in Cyclin D1 and cdk4/6 expression leading to apoptotic cell death in hippocampal and cortical neurons has been reported recently [51]. BDNF deprivation has been reported to show upregulated Cyclin D1 expression and induction of apoptosis in cerebellar granule cells [52]. Both PCNA and Cyclin D1 expression was found to be upregulated in mutant mouse models of trophic factors [53]. B-TCE pretreatment suppressed the increase in Cyclin D1 and PCNA expression after exposure to glutamate, thus, may prevent the glutamate-induced DNA damage and apoptosis induction. The nuclear condensation (as evident from DAPI staining), mitochondrial dysfunction (Mitoctracker staining) and cell cycle deregulation (evident from Cyclin D1 and PCNA) indicated the induction of apoptosis after glutamate treatment. B-TCE pretreatment significantly upregulated Bcl-xL expression (*p* ≤ 0.01) and suppressed apoptosis induced by glutamate exposure in primary cerebellar neurons. Previous reports have also suggested that oxidative stress due to high concentration of glutamate induces mitochondrial dysfunction which results in cytochrome c release and activation of downstream molecules involved in apoptosis induction [31]. Overexpression of Bcl-xL, an anti-apoptotic protein of the Bcl-2 family was also shown to delay cytochrome c release from mitochondria in response to Bax in Human embryonic kidney (293 T) cells [54]. In view of these previous reports and our current observations, it may be suggested that upregulated expression of Bcl-xL mitigated the apoptosis induction by suppressing cytochrome c release from mitochondria and activation of downstream activation of apoptosis pathway. A slight increase in Bcl-xL expression in B-TCE alone group may be helping the cells to preserve axonal morphology. Increase in Bcl-xL has also been reported to play important role in functional adaptation and enhanced lifespan of cells by preventing apoptosis as well as by preservation of axonal morphology [55, 56].

We further observed that glutamate exposure downregulated expression of PSA-NCAM and NCAM, whereas, B-TCE pretreatment prevented these changes and maintained the expression of these plasticity proteins to near-control levels. Cell adhesion molecules play important role in cell-cell interactions, migration, plasticity, regeneration and repair [57]. NCAM is a potential neuroprotective protein, and its enhanced expression suggests that B-TCE exerts neuroprotection by upregulating the expression of these neuroprotective plasticity proteins [58, 59]. Polysialated form of NCAM is a characteristic marker of developing, migrating neurons and synaptogenesis of nervous tissue. Application of PSA-NCAM was found to reduce excitotoxic death of cultured hippocampal neurons due to glutamate exposure [60]. In a previous interventional study from our lab, dietary restriction was seen to exert neuroprotection against kainic acid-induced toxicity by upregulating NCAM and PSA-NCAM expression [56]. Further, the neuroprotective activity of Ashwagandha leaf water extract against glutamate-induced excitotoxicity was also reported to upregulate PSA-NCAM and NCAM expression [46]. Neurite outgrowth and migration of glutamate exposed primary cerebellar neurons in the lesioned area was promoted by B-TCE pretreatment which may be attributed to upregulated PSA-NCAM and NCAM expression. Sprouting from organotypic cultures of hippocampal slices in lesion-induced neurite outgrowth model was associated with a pronounced expression of PSA-NCAM [57]. Both of these cell adhesion molecules participate in neurite outgrowth and synaptogenesis and their downregulated expression resulted in glutamate-induced dendritic atrophy in hippocampal neurons [61]. The neurite outgrowth and migration were also accompanied by enhanced MMP-2 and MMP-9 expression which was maintained by B-TCE pretreatment, whereas, glutamate treatment significantly downregulated MMP-2 expression (*p* ≤ 0.01). MMP-2 and MMP-9 are major MMPs which play important role in cellular motility and neurite outgrowth across matrix under different pathological and physiological conditions [62]. Depletion of MMP-2 and MMP-9 from culture conditioned media was reported to abolish neurite outgrowth and axonal regeneration from cortical neurons [63]. Based on these observations, it may be suggested that B-TCE pretreatment of primary cerebellar neurons before glutamate exposure promoted migration, neurite outgrowth and enhanced neural plasticity.

We also attempted to characterize eight peaks corresponding to magnoflorine, palmatine, norcoclaurine, cordifolioside A, oblongine, tetrahydropalmatine, 11-hydroxy mustakone and tinocoriside which belong to alkaloids, glycosides and sesquiterpenoids. Our findings are in line with previous reports suggesting cordifolioside A and tinocordiside as active constituents of n-butanol fraction of *T. cordifolia* stem extract [20, 21]. Alkaloids magnoflorine and palmatine were also reported to present in n-butanol fraction of *T. cordifolia* stem extract. The neuroprotective and immune-modulatory activity has been attributed to the presence of alkaloids and glycosides in *T. cordifolia* [17, 63]. Cordifolioside A and B are known to exhibit immunostimulating activity [64, 65]. Further, cordifolioside A has been reported to exert radio and cytoprotective activity [21], whereas, tinocordiside exerted cytotoxicity against cancerous KB and Siha cell lines [66]. Alkaloids palmatine, magnoflorine are reported to possess different biological activities such as anti-cancer, anti-glycemic, whereas, sesquiterpene 11-hydroxymuskatone induced significant proliferation of splenocyte, thus acting as immunomodulatory compound [63]. Levo-tetrahydropalmatine has been reported as a dopamine receptor antagonist, used against drug self-administration and reinstatement behaviour [67, 68]. Presence of dopamine receptor antagonists in *T. cordifolia,* therefore, may explain the basis of anxiolytic, anti-psychotic and neuroprotective effect of extract reported earlier [15, 16, 67, 68].

## Conclusions

**Fig. 7:**
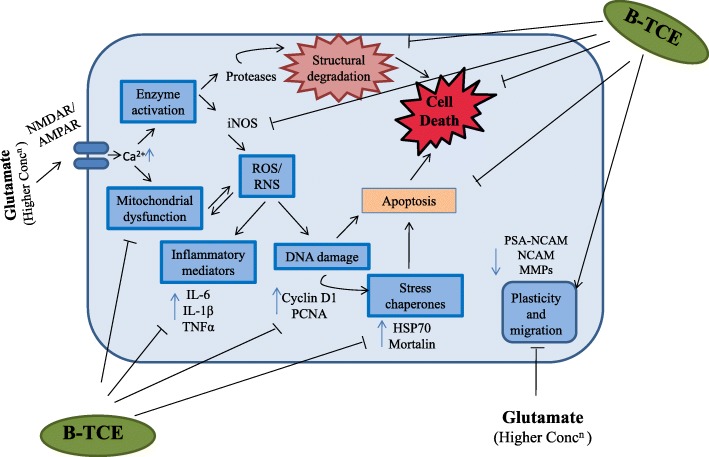
Representative graphical image presenting the underlying mechanism of glutamate-induced excitotoxicity and how B-TCE pretreatment protects primary cerebellar neurons by modulating the expression of different molecular effectors. Overstimulation of glutamate receptors induced rise in intracellular calcium which leads to mitochondrial dysfunction and activation of enzymes which further results in ROS/RNS generation and structural degradation of neurons. ROS/RNS induce upregulation of inflammatory mediators, DNA damage and stress chaperones thus causing apoptosis and neurodegeneration. In the present study B-TCE pretreatment inhibited glutamate-induced structural degradation (morphometric studies, MAP-2, GAP-43, NF200), mitochondrial dysfunction (Mitotracker green FM), suppressed NO generation (iNOS), secretion of pro-inflammatory cytokines (TNF-α, IL-6 and IL-1β), DNA damage (Cyclin D1 and PCNA) and stress proteins expression (HSP70 and Mortalin), thus inhibited apoptosis induction and neurodegeneration. B-TCE also promoted neuronal plasticity and migration (PSA-NCAM, NCAM expression)

The aim of current study was to investigate the potential benefits of B-TCE in amelioration of glutamate-induced excitotoxicity and it provides the first evidence of neuroprotective and neuroregenerative potential of *T. cordifolia*. Earlier, our lab has tested the crude TCE for its anticancer potential which may be attributed to its hexane and chloroform fraction (effective at much lower concentration), whereas, Butanol fraction showed strong neuroprotective and neuroregenerative potential. Current data may suggest that B-TCE exerted neuroprotection against glutamate-induced excitotoxicity by modulating different pathways such as neuronal differentiation, homeostasis and apoptosis. B-TCE pretreatment prevented glutamate-induced insults on neuronal integrity, promoted neurite outgrowth and cell migration. It also suppressed glutamate-induced onset of inflammation and stress chaperones expression (Fig. 7). The neuroprotective activity may be attributed to immunomodulatory compounds present in this fraction, however, isolation and characterization of single active compounds are being planned in the future study. These findings bestow a stepping stone towards future research aiming to investigate the role of *T. cordifolia* as a candidate for herbal based neuroprotective and neuroregenerative approach against neurodegenerative diseases. B-TCE could be used as a safe and effective non-palliative therapeutic candidate against neurodegenerative diseases.

## Additional file

Additional file 1: **Figure S1-S12.** Supporting data to dose selection, cellular and nuclear morphology and spectra corresponding to UPLC-MS. (PPTX 1379 kb)

## Abbreviations

6-OHDA: 6-Hydroxydopamine
AP-1: Activator protein-1
Bcl-xL: B-cell lymphoma-extra large
BDNF: Brain-derived neurotrophic factor
B-TCE: Butanol extract of *T. cordifolia*
Chl-TCE: Chloroform extract of *T. cordifolia*
DAPI: 4′,6-diamidino-2-phenylindole
ELISA: Enzyme-linked immunosorbent assay
GAP-43: Growth associated protein-43
GAPDH: Glyceraldehyde 3-phosphate dehydrogenase
Hex-TCE: Hexane extract of *T. cordifolia*
HSP70: Heat shock protein 70
IL-1β: Interleukin-1beta
IL-6: Interleukin-6
MAP-2: Microtubule-associated protein 2
MAPK: Mitogen associated protein kinase
MMP: Matrix metalloproteinases
NCAM: Neural cell adhesion molecule
NF200: Neurofilament 200
NF-κB: Nuclear factor-kappa beta
NOS: Nitric oxide synthase
PCNA: Proliferating cell nuclear antigen
PSA-NCAM: Polysialic acid-NCAM
ROS/RNS: Reactive oxygen/ nitrogen species
TNFα: Tumor necrosis factor alpha
UPLC-MS: Ultra performance liquid chromatography-mass spectrometry
