# Supplementary material for: Tinospora cordifolia as a potential neuroregenerative candidate against glutamate induced excitotoxicity: an in vitro perspective
Source: BMC Complement Altern Med. 2018 Oct 1;18:268. doi: 10.1186/s12906-018-2330-6 (PMC6167833; doi:10.1186/s12906-018-2330-6)
Supplement: Supplementary file 1 — Figure S1-S12. Supporting data to dose selection, cellular and nuclear morphology and spectra corresponding to UPLC-MS. (PPTX 1379 kb) [file 12906_2018_2330_MOESM1_ESM.pptx]

## Slide 1
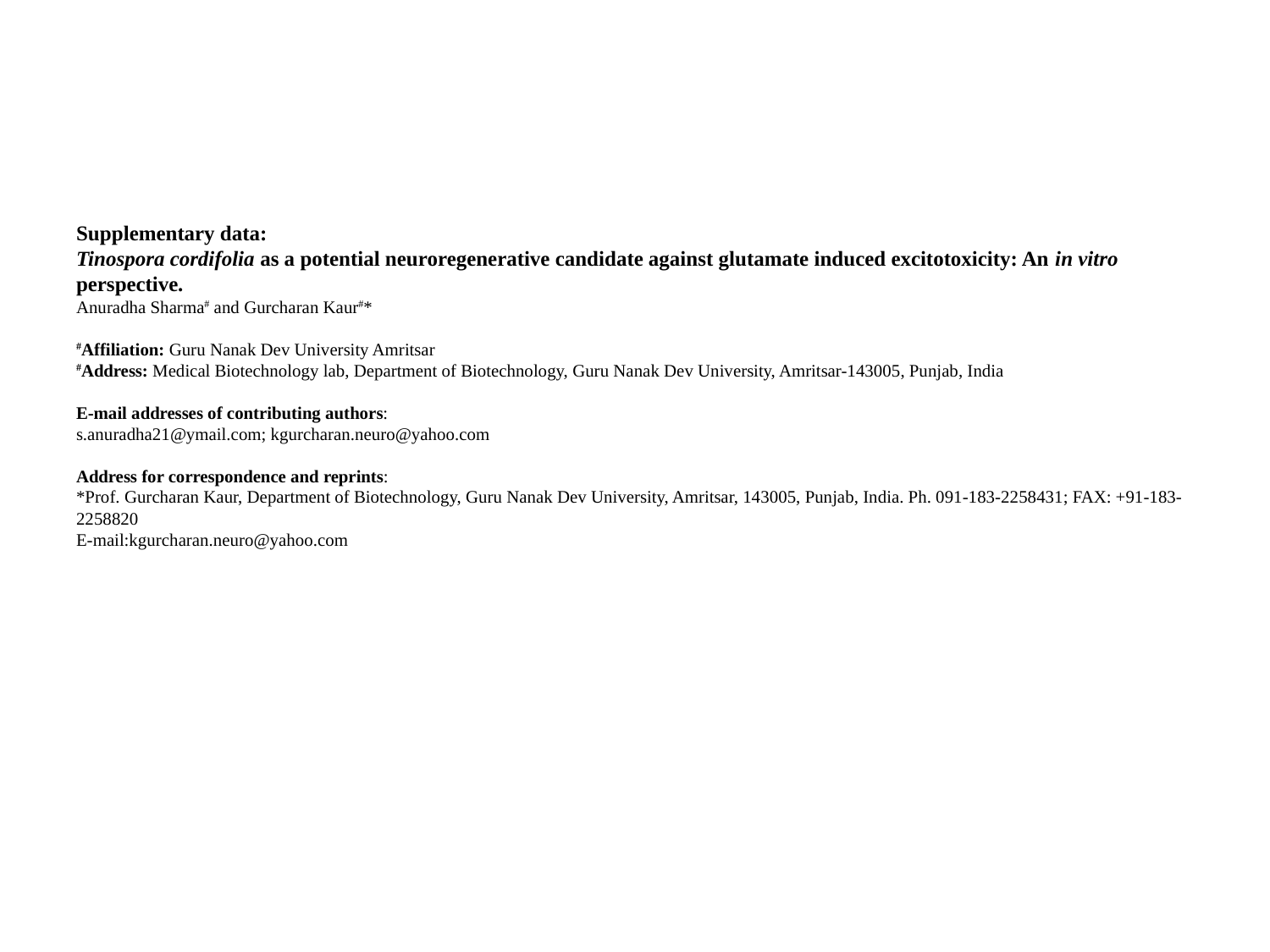

Supplementary data:
Tinospora cordifolia as a potential neuroregenerative candidate against glutamate induced excitotoxicity: An in vitro perspective.
Anuradha Sharma# and Gurcharan Kaur#*
#Affiliation: Guru Nanak Dev University Amritsar
#Address: Medical Biotechnology lab, Department of Biotechnology, Guru Nanak Dev University, Amritsar-143005, Punjab, India
E-mail addresses of contributing authors:
s.anuradha21@ymail.com; kgurcharan.neuro@yahoo.com
Address for correspondence and reprints:
*Prof. Gurcharan Kaur, Department of Biotechnology, Guru Nanak Dev University, Amritsar, 143005, Punjab, India. Ph. 091-183-2258431; FAX: +91-183-2258820
E-mail:kgurcharan.neuro@yahoo.com

## Slide 2
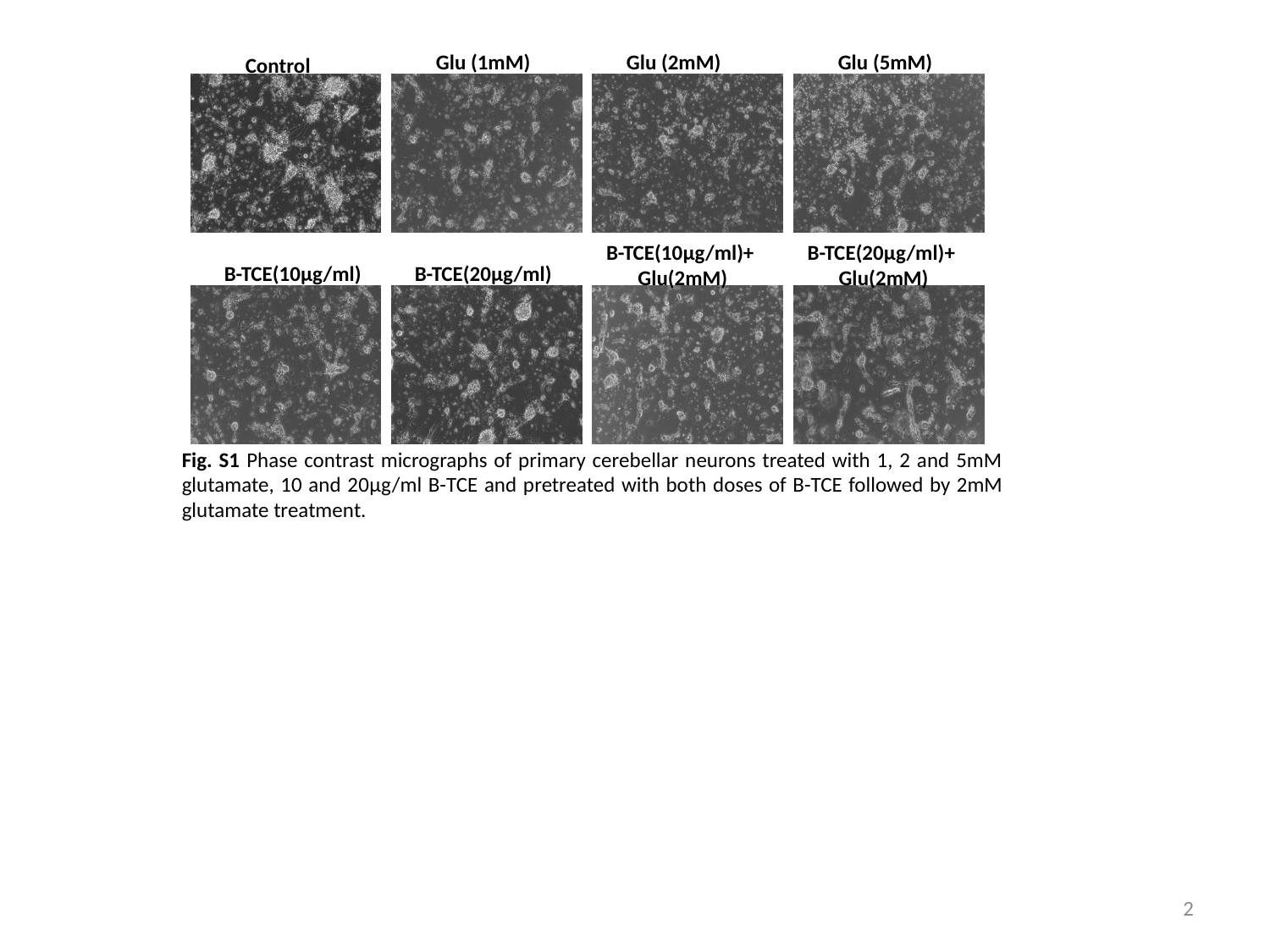

Glu (1mM)
Glu (2mM)
Glu (5mM)
Control
B-TCE(10μg/ml)+
Glu(2mM)
B-TCE(20μg/ml)+
Glu(2mM)
B-TCE(10μg/ml)
B-TCE(20μg/ml)
Fig. S1 Phase contrast micrographs of primary cerebellar neurons treated with 1, 2 and 5mM glutamate, 10 and 20μg/ml B-TCE and pretreated with both doses of B-TCE followed by 2mM glutamate treatment.
2

## Slide 3
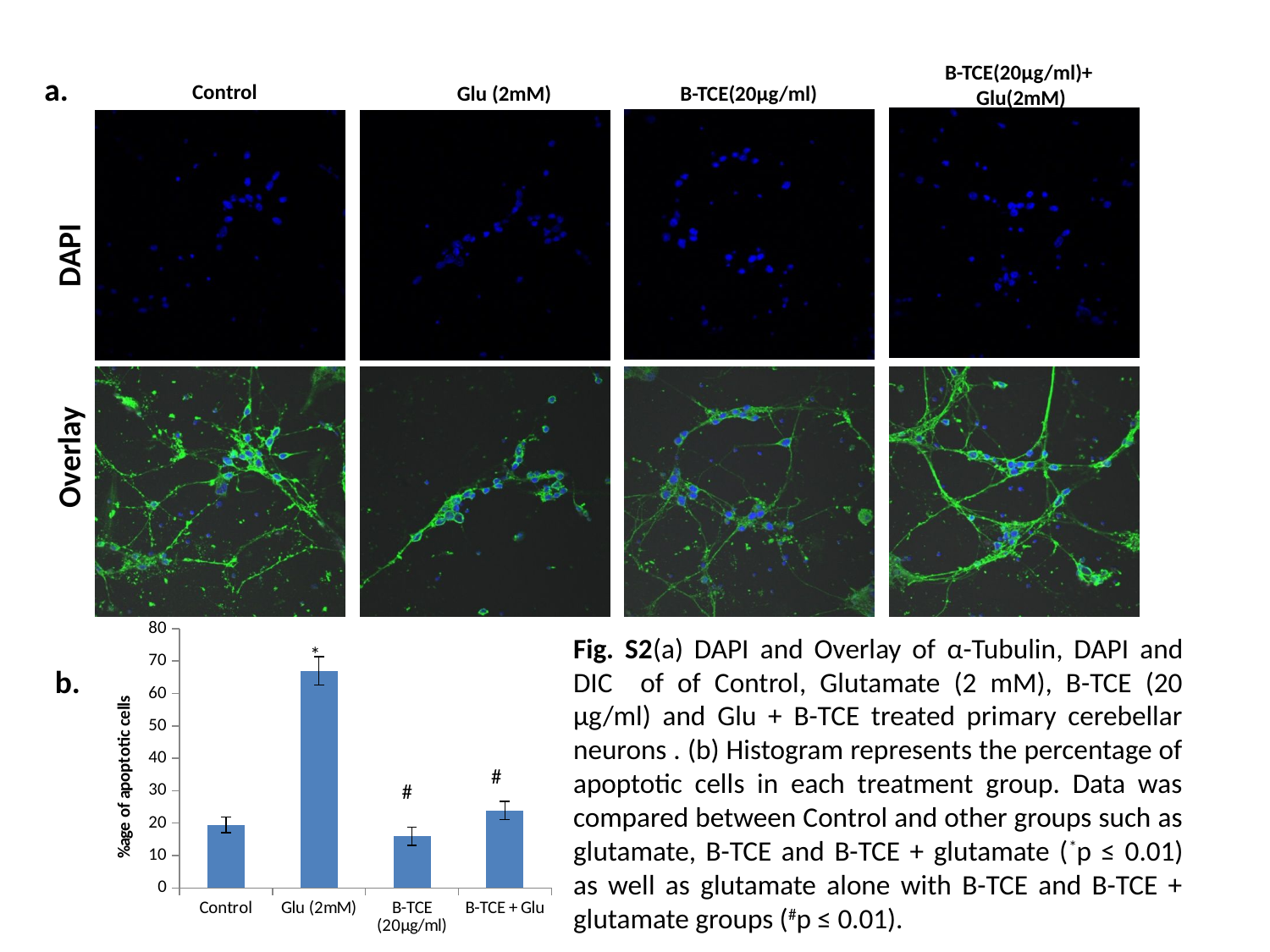

B-TCE(20μg/ml)+
Glu(2mM)
a.
Control
Glu (2mM)
B-TCE(20μg/ml)
DAPI
Overlay
### Chart
| Category | |
|---|---|
| Control | 19.5 |
| Glu (2mM) | 67.0 |
| B-TCE (20µg/ml) | 15.9 |
| B-TCE + Glu | 23.9 |*
#
#
Fig. S2(a) DAPI and Overlay of α-Tubulin, DAPI and DIC of of Control, Glutamate (2 mM), B-TCE (20 μg/ml) and Glu + B-TCE treated primary cerebellar neurons . (b) Histogram represents the percentage of apoptotic cells in each treatment group. Data was compared between Control and other groups such as glutamate, B-TCE and B-TCE + glutamate (*p ≤ 0.01) as well as glutamate alone with B-TCE and B-TCE + glutamate groups (#p ≤ 0.01).
b.

## Slide 4
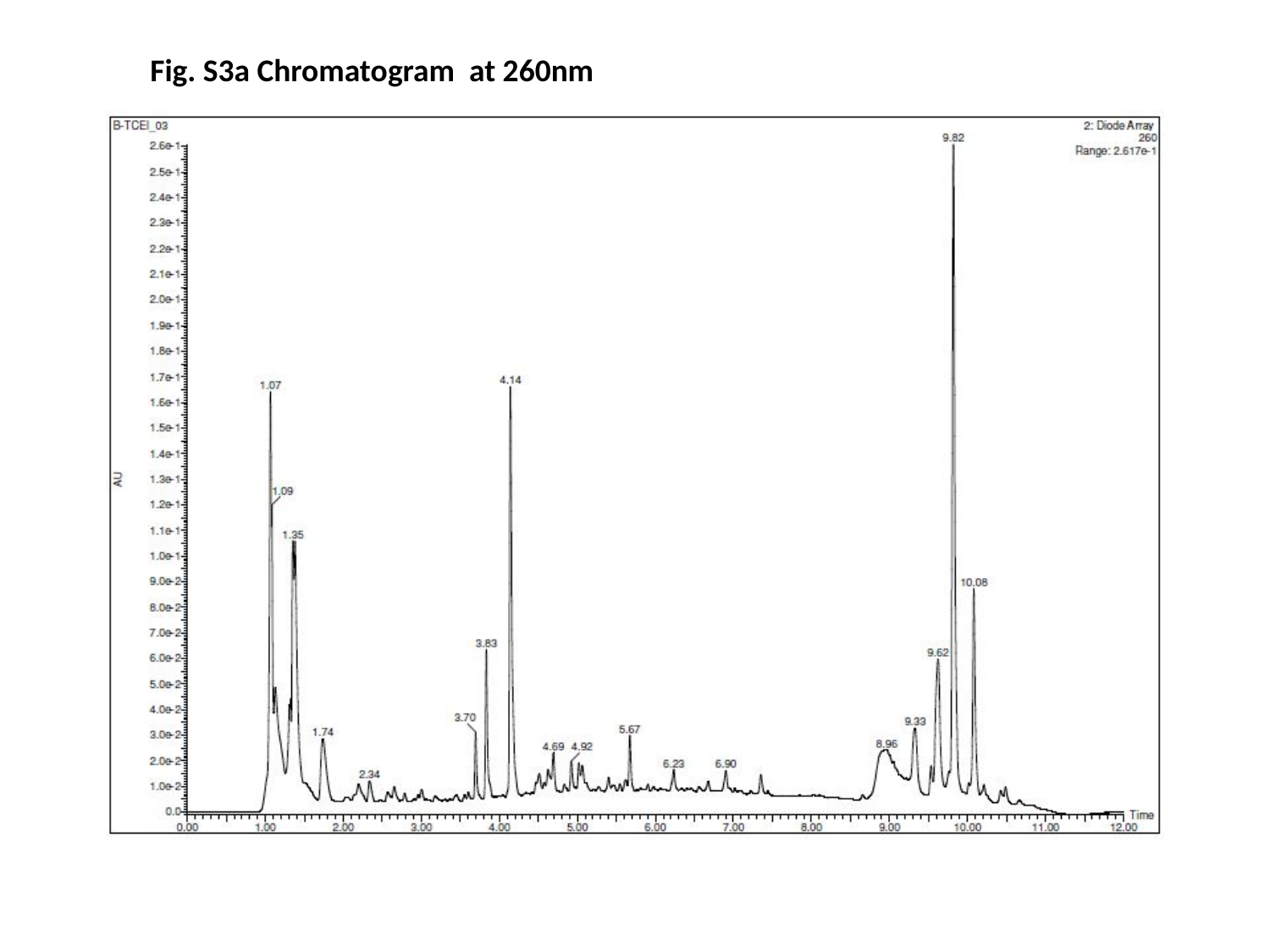

Fig. S3a Chromatogram at 260nm

## Slide 5
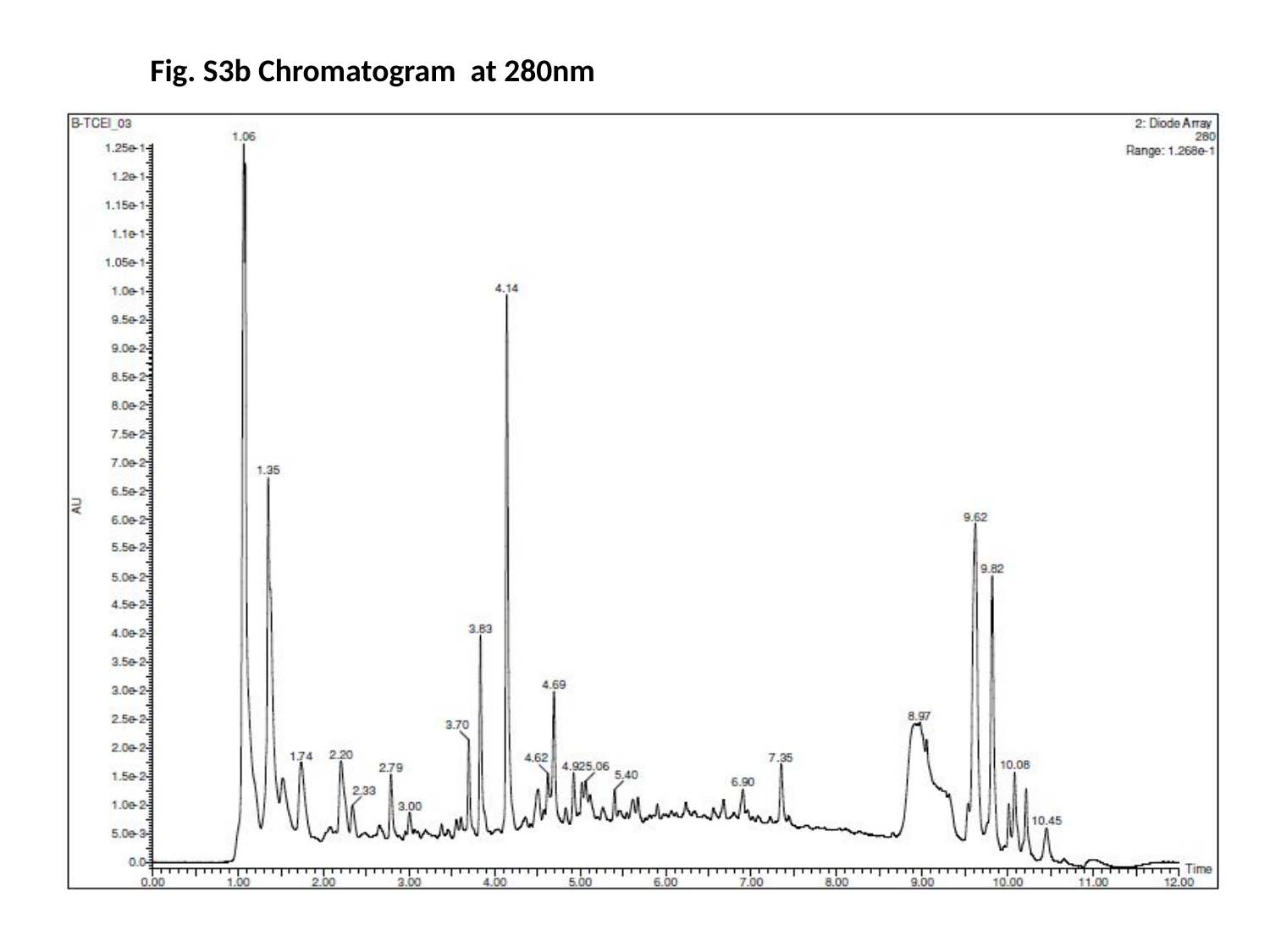

Fig. S3b Chromatogram at 280nm

## Slide 6
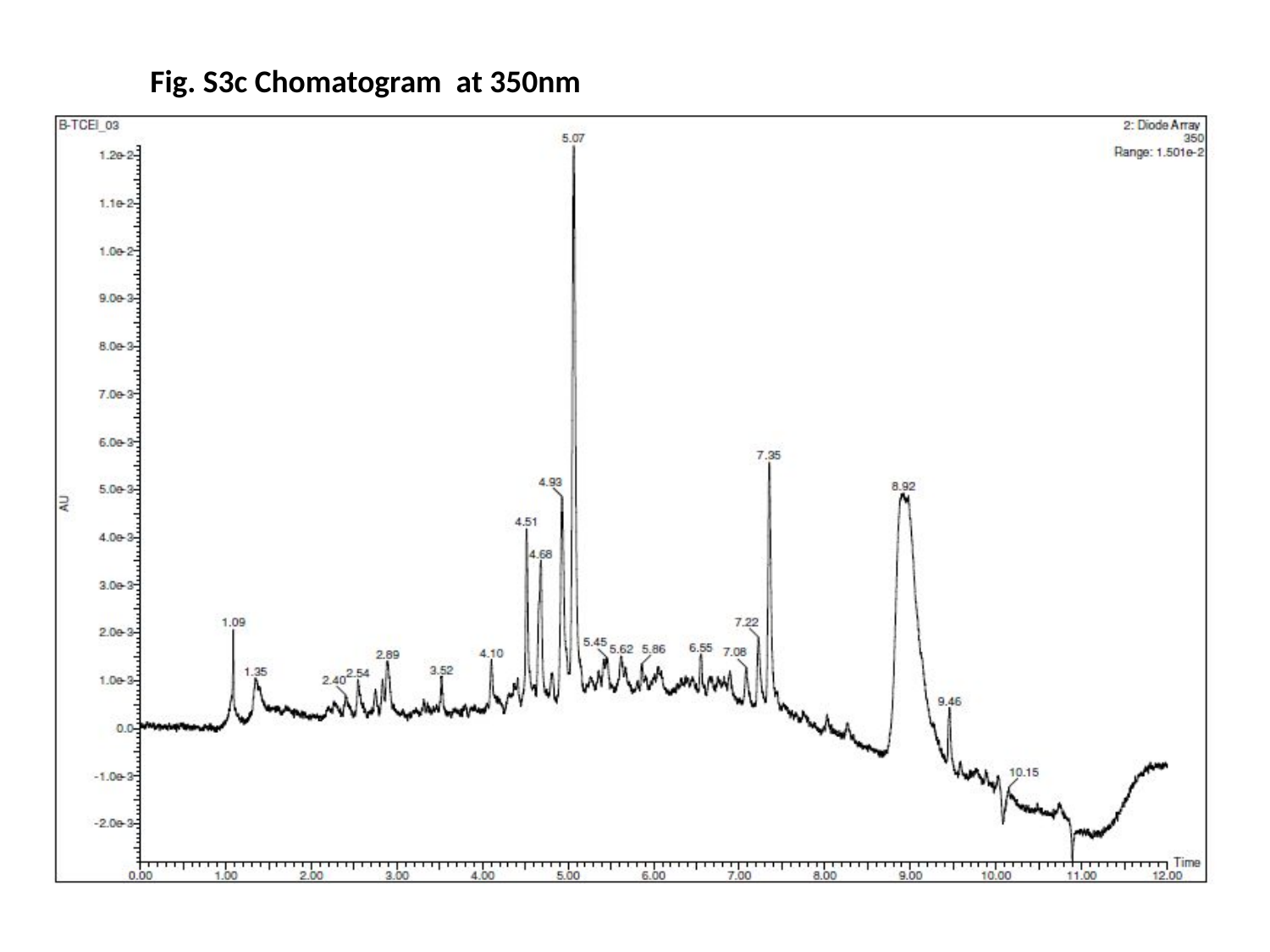

Fig. S3c Chomatogram at 350nm

## Slide 7
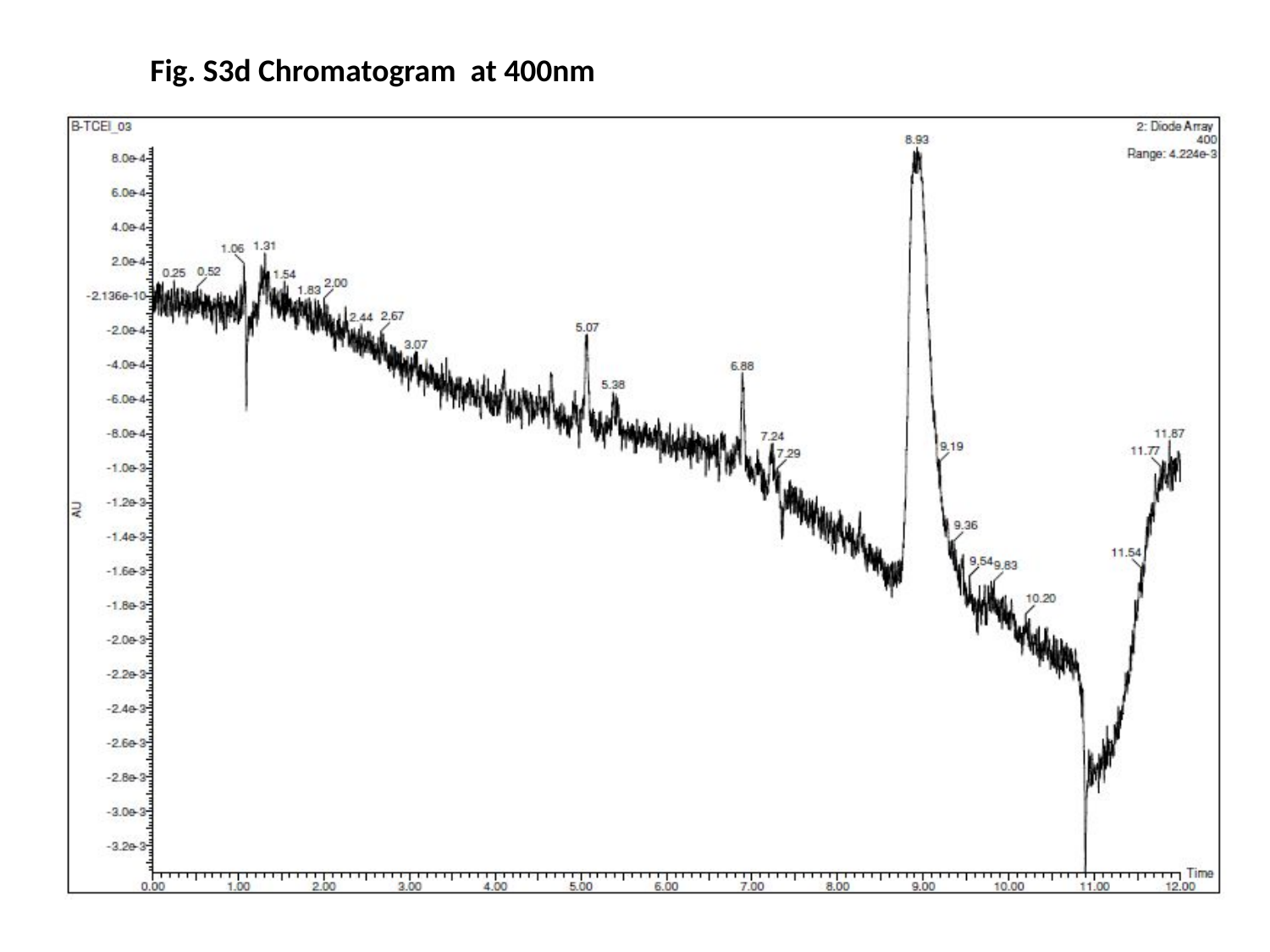

Fig. S3d Chromatogram at 400nm

## Slide 8
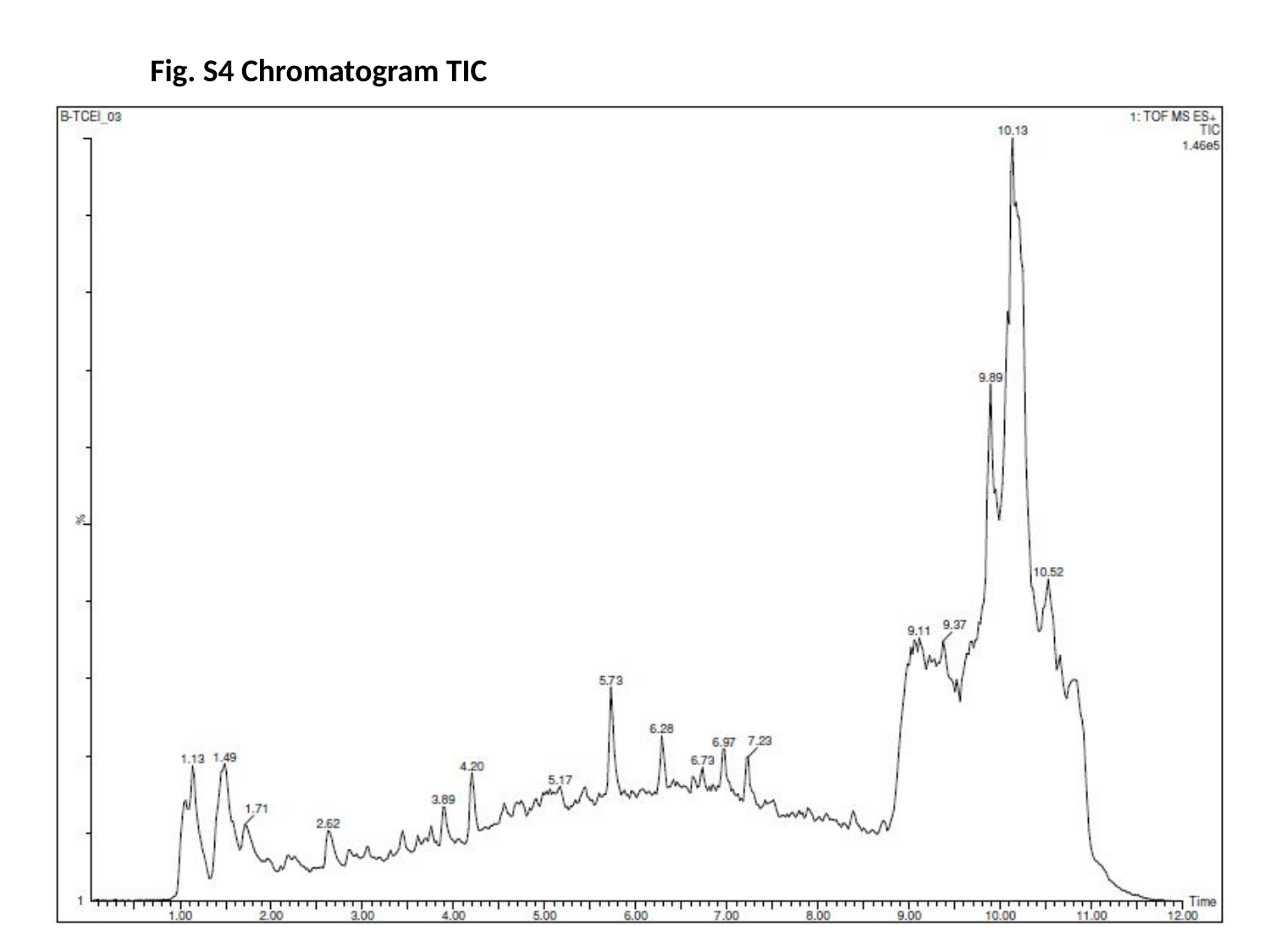

Fig. S4 Chromatogram TIC

## Slide 9
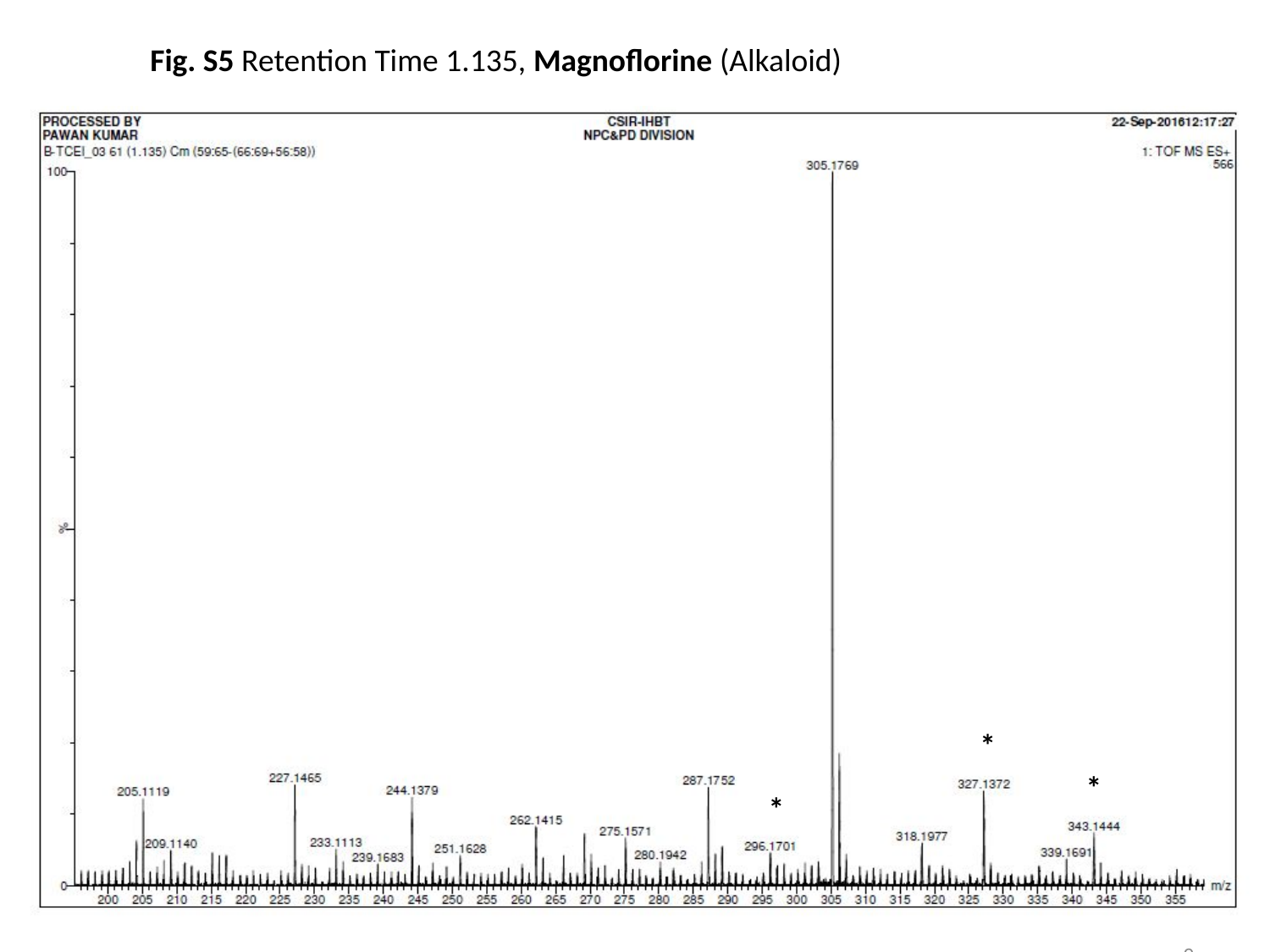

Fig. S5 Retention Time 1.135, Magnoflorine (Alkaloid)
*
*
*
9

## Slide 10
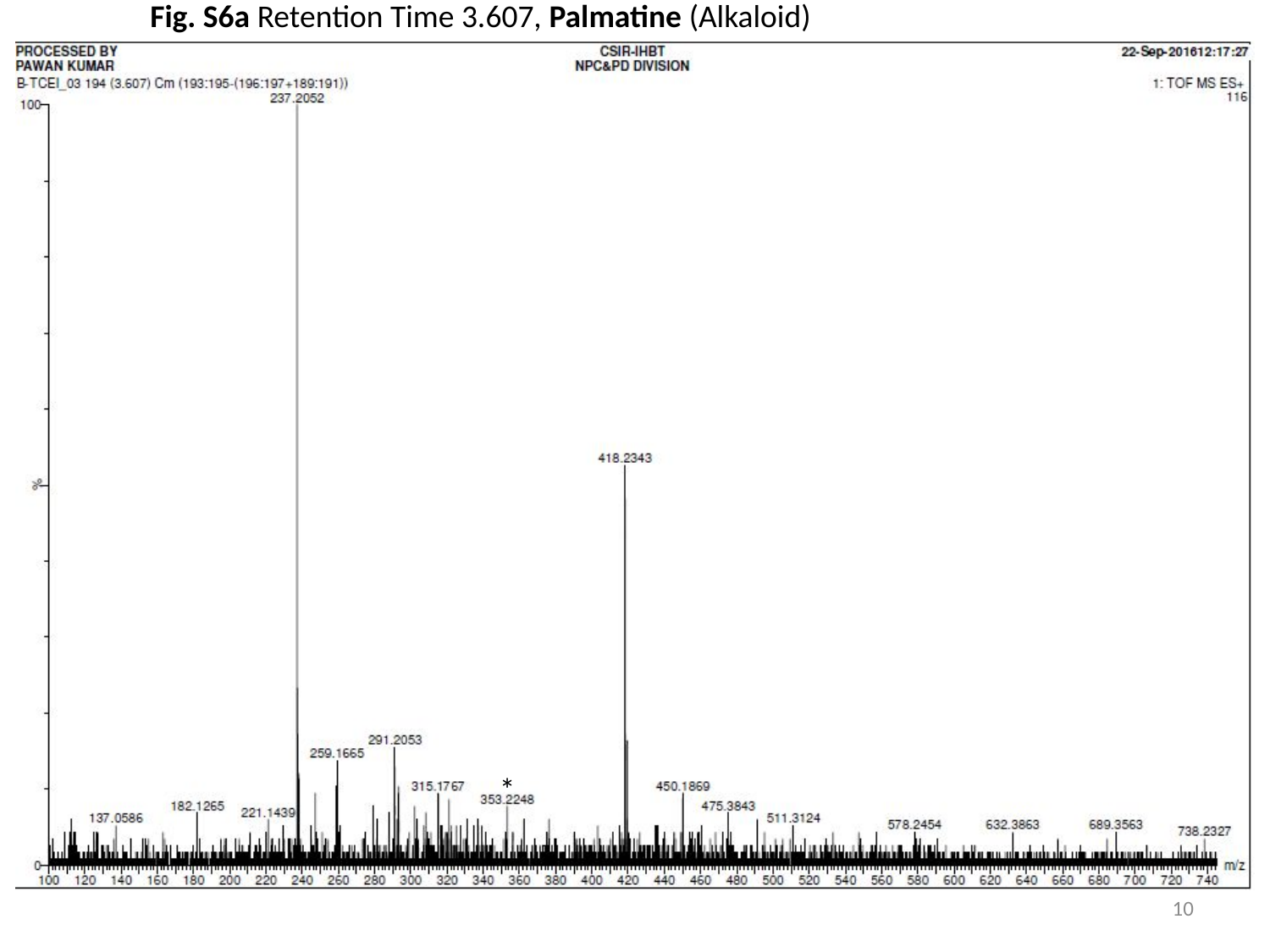

Fig. S6a Retention Time 3.607, Palmatine (Alkaloid)
*
10

## Slide 11
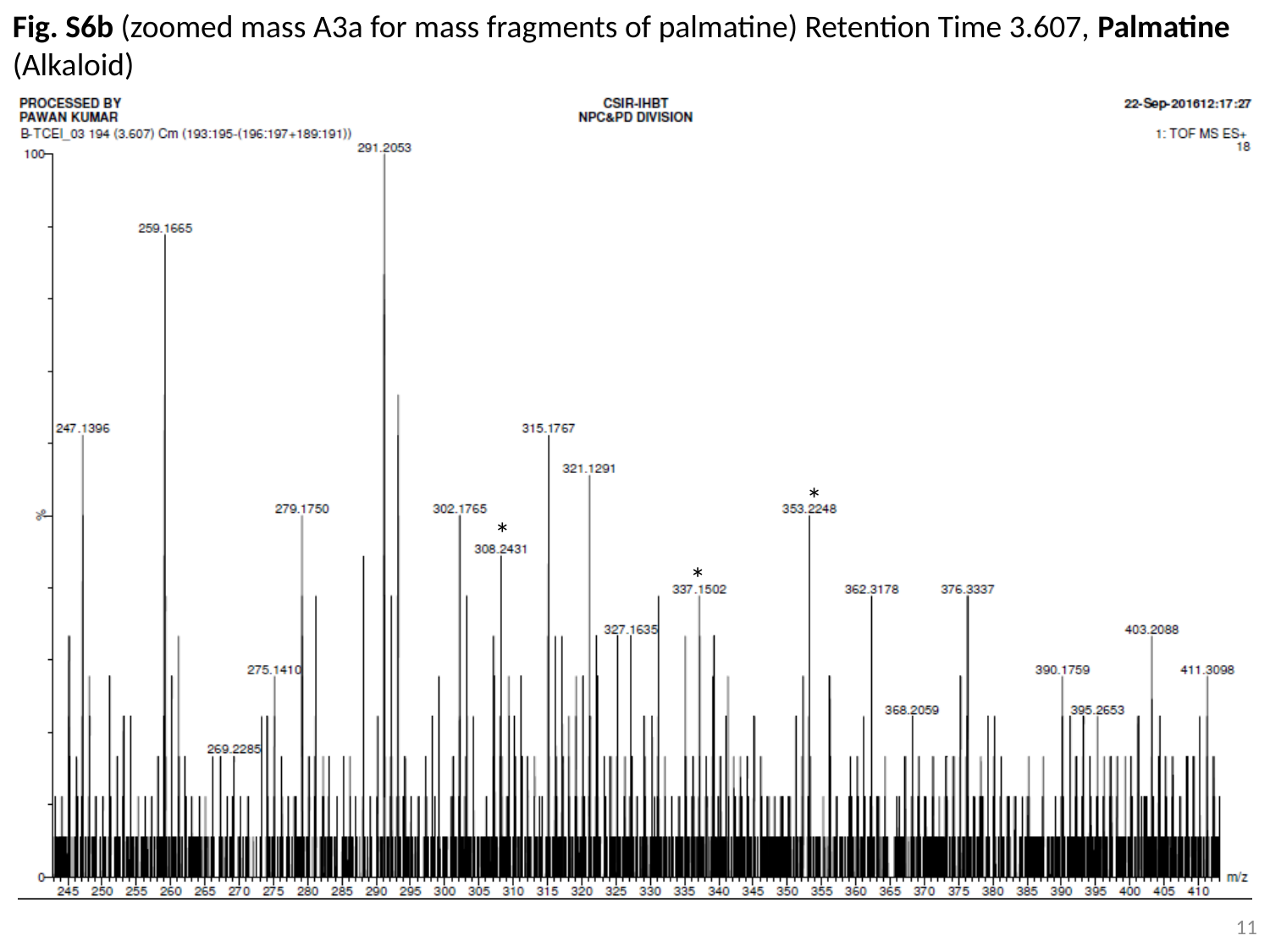

Fig. S6b (zoomed mass A3a for mass fragments of palmatine) Retention Time 3.607, Palmatine (Alkaloid)
*
*
*
11

## Slide 12
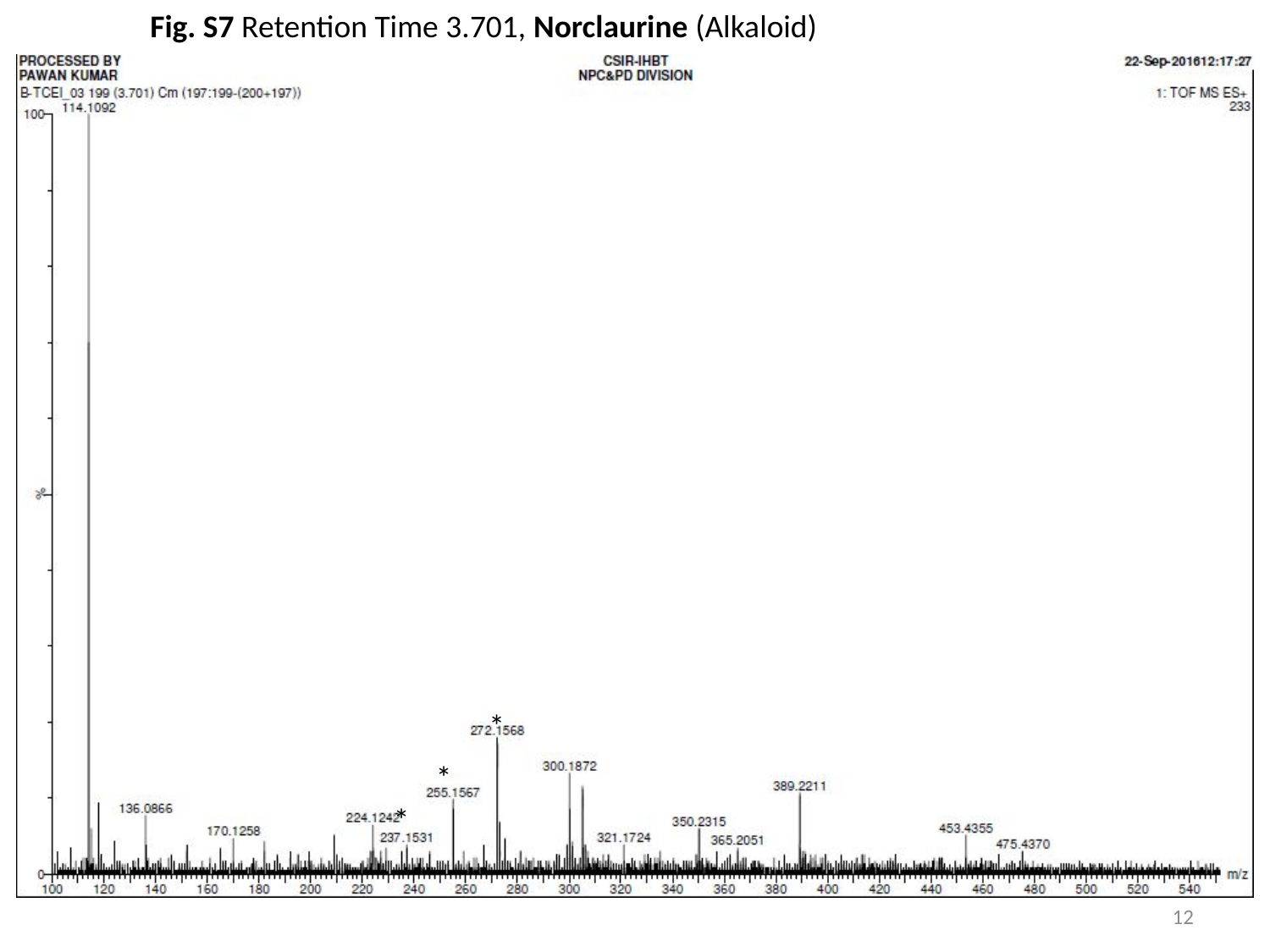

Fig. S7 Retention Time 3.701, Norclaurine (Alkaloid)
*
*
*
12

## Slide 13
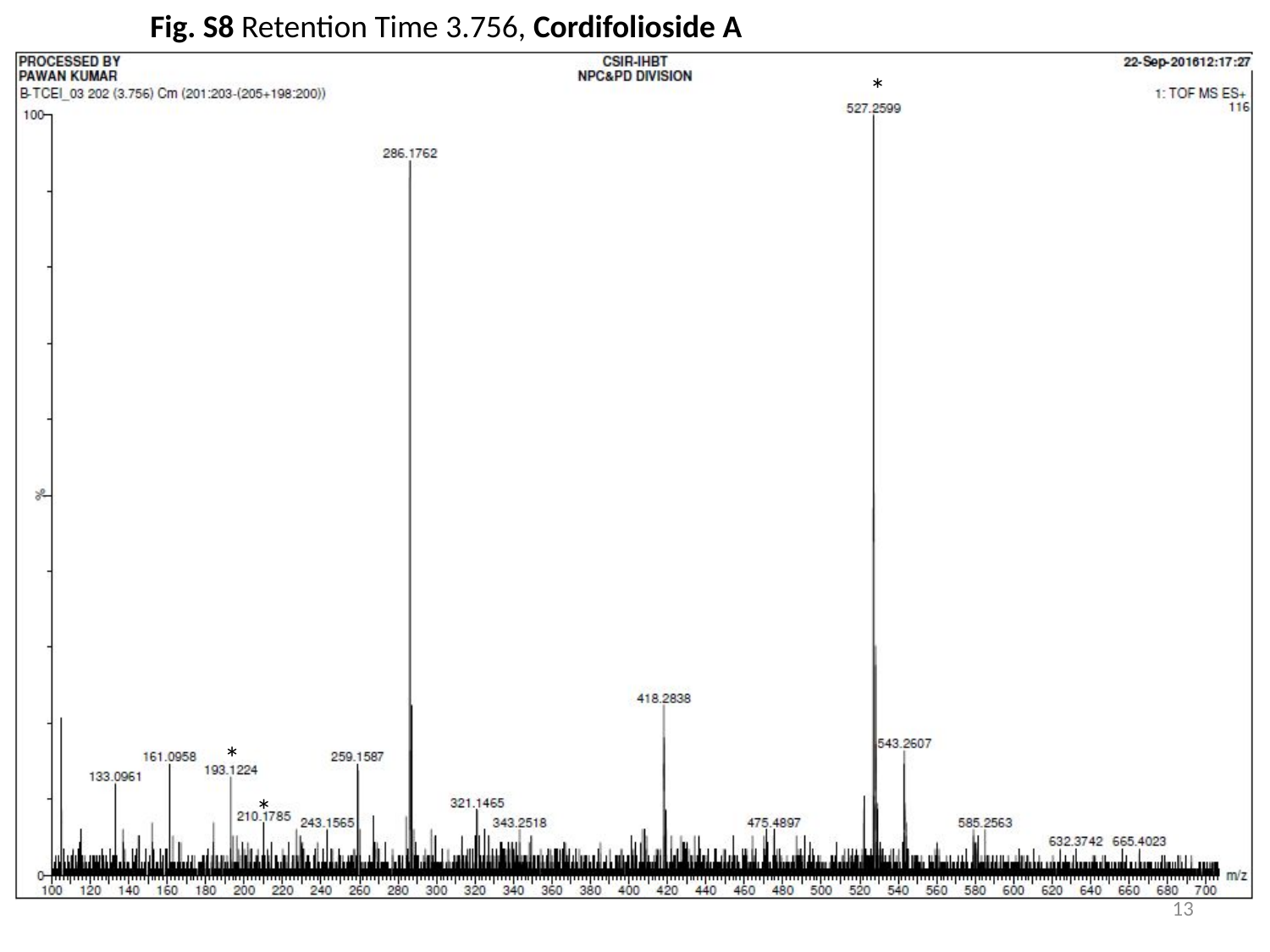

Fig. S8 Retention Time 3.756, Cordifolioside A
*
*
*
13

## Slide 14
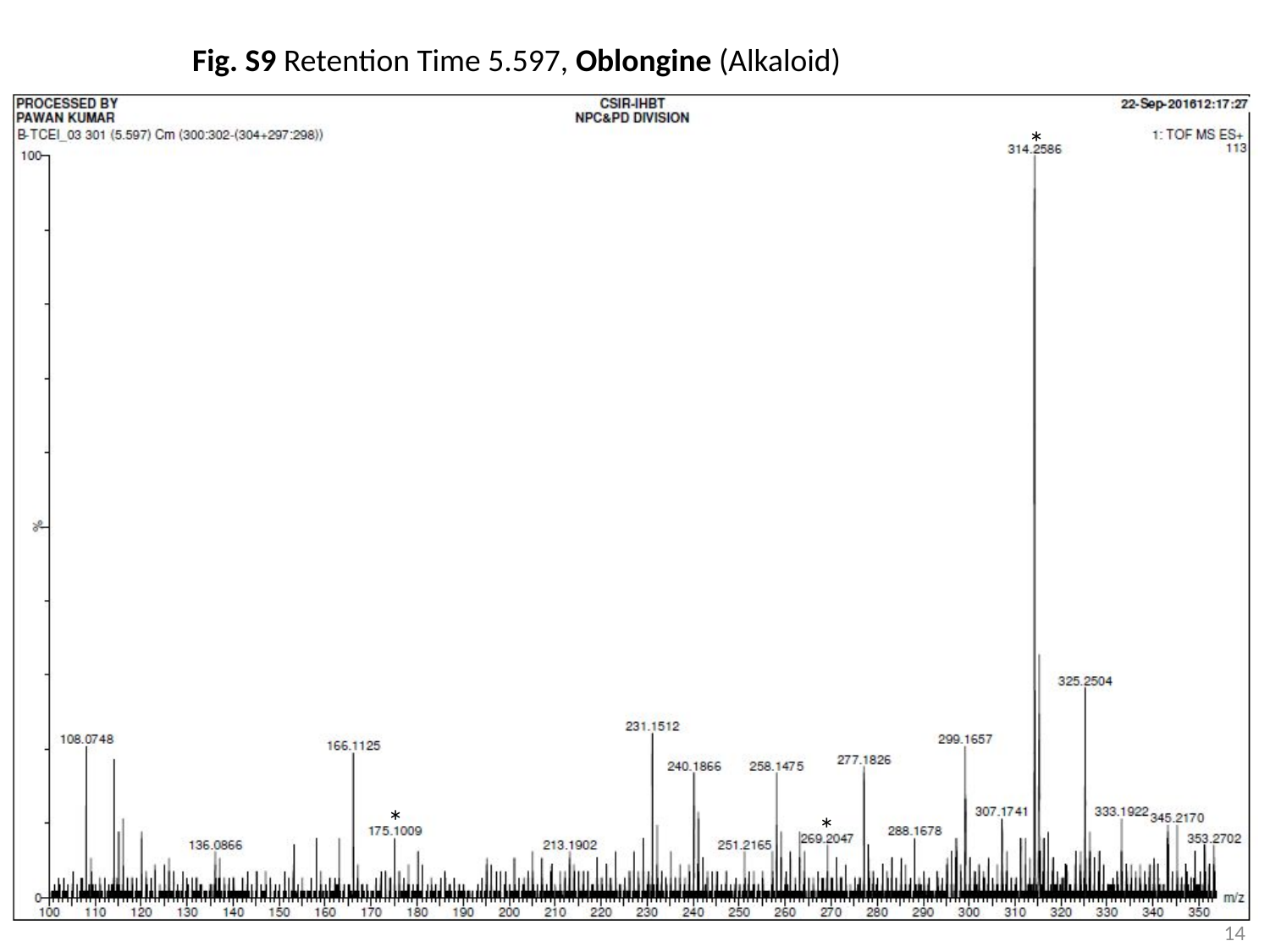

Fig. S9 Retention Time 5.597, Oblongine (Alkaloid)
*
*
*
14

## Slide 15
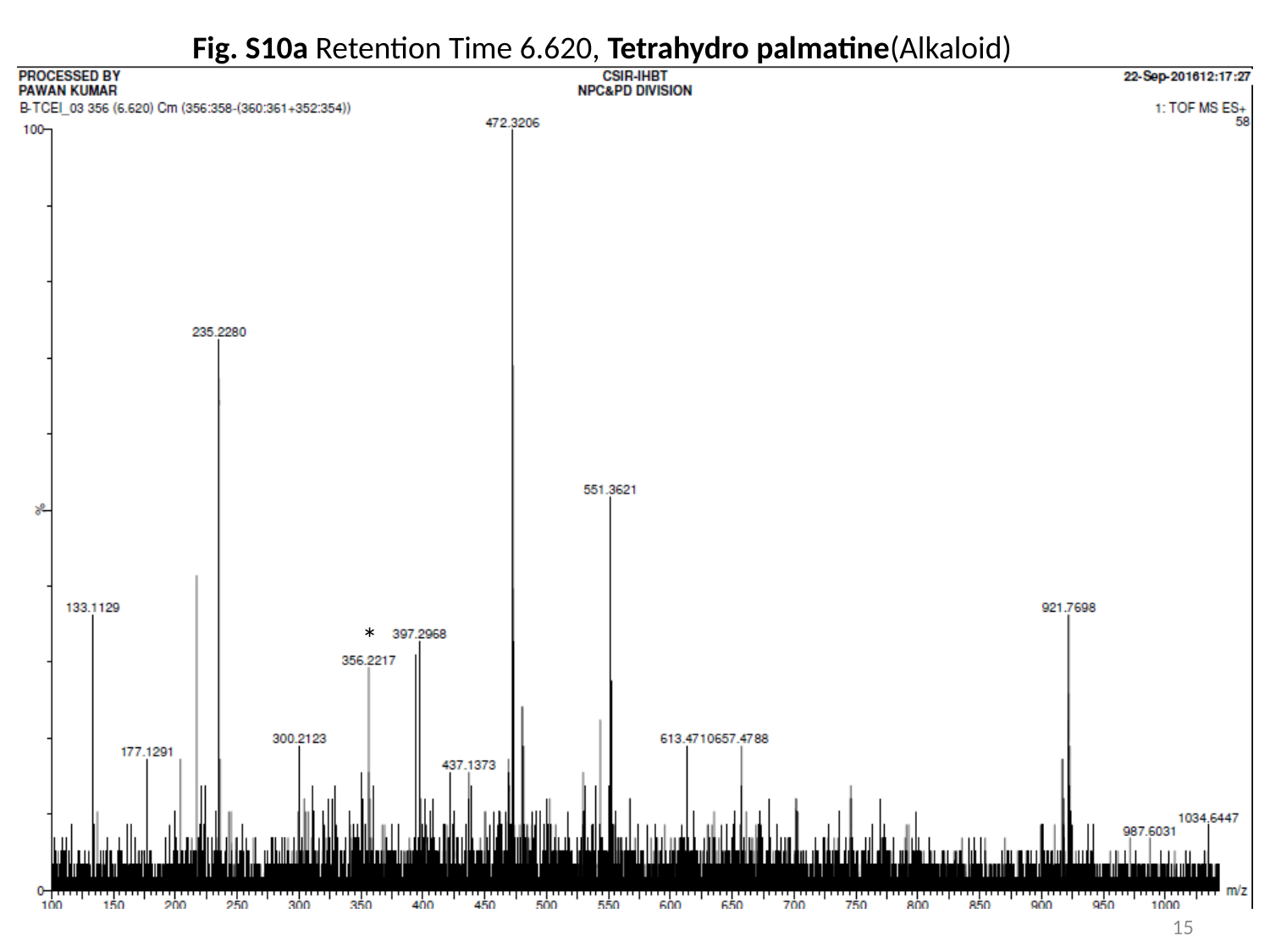

Fig. S10a Retention Time 6.620, Tetrahydro palmatine(Alkaloid)
*
15

## Slide 16
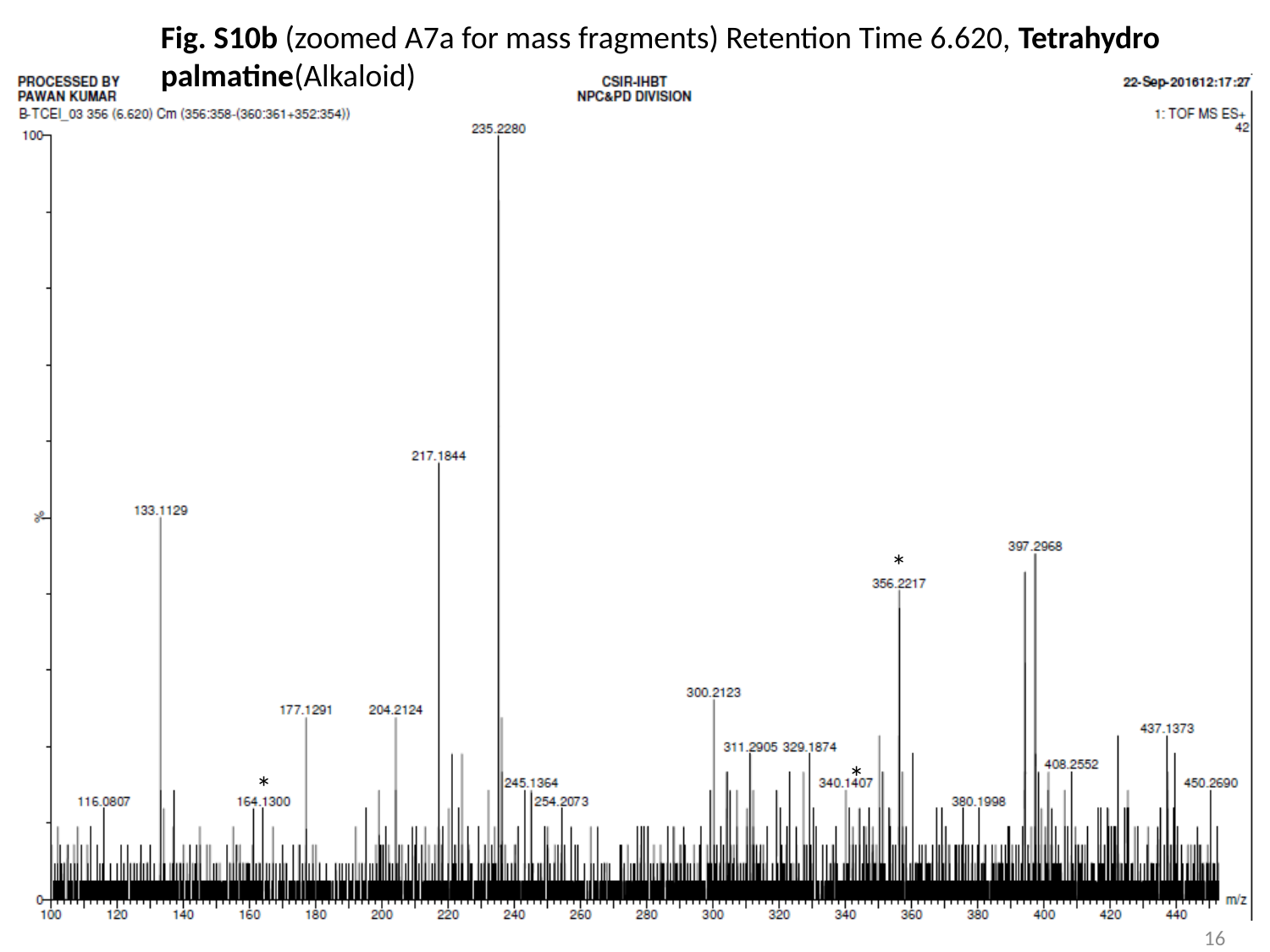

Fig. S10b (zoomed A7a for mass fragments) Retention Time 6.620, Tetrahydro palmatine(Alkaloid)
*
*
*
16

## Slide 17
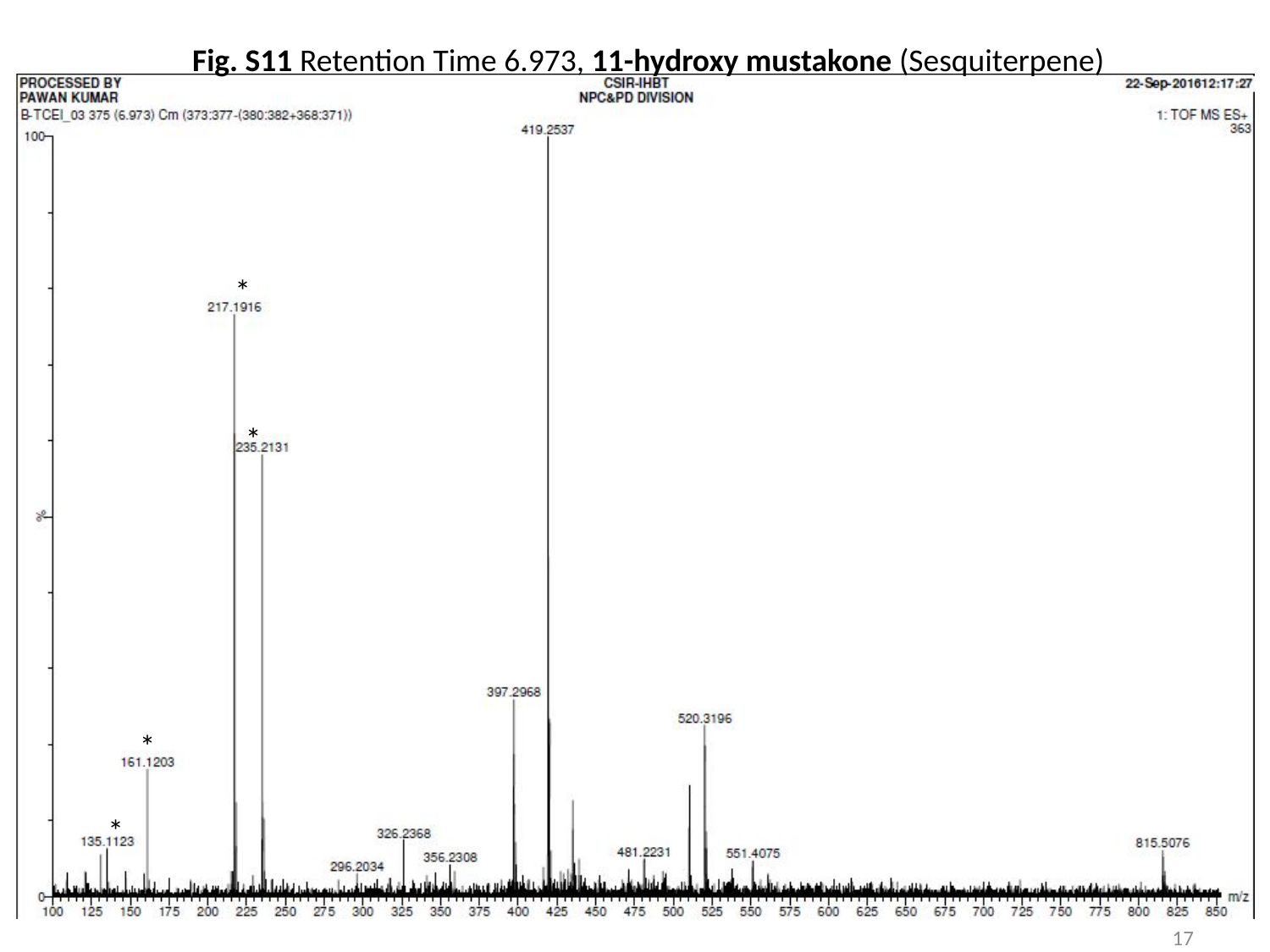

Fig. S11 Retention Time 6.973, 11-hydroxy mustakone (Sesquiterpene)
*
*
*
*
17

## Slide 18
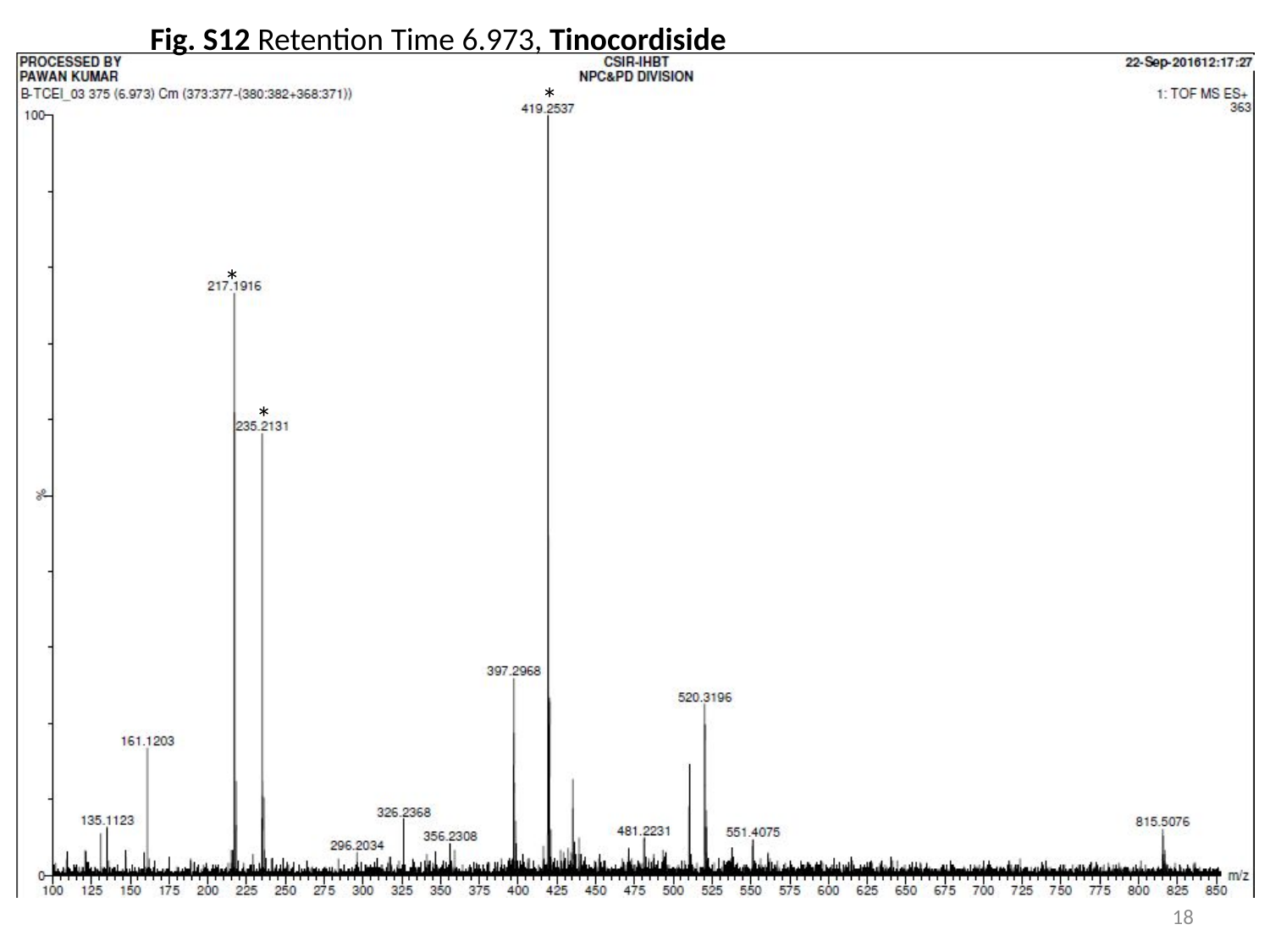

Fig. S12 Retention Time 6.973, Tinocordiside
*
*
*
18
